# An Uncommon Phosphorylation Mode Regulates the Activity and Protein Interactions of *N*-Acetylglucosamine Kinase

**DOI:** 10.1021/jacs.4c03069

**Published:** 2024-05-11

**Authors:** Arif Celik, Ida Beyer, Dorothea Fiedler

**Affiliations:** † Leibniz-Forschungsinstitut für Molekulare Pharmakologie (FMP), Robert-Rössle-Straße 10, 13125 Berlin, Germany; ‡ Institut für Chemie, Humboldt-Universität zu Berlin, Brook-Taylor-Str. 2, 12489 Berlin, Germany

## Abstract

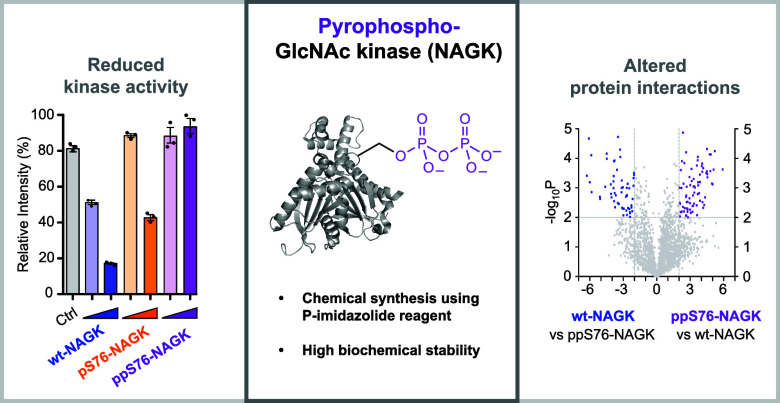

While the function of protein phosphorylation in eukaryotic cell signaling is well established, the role of a closely related modification, protein pyrophosphorylation, is just starting to surface. A recent study has identified several targets of endogenous protein pyrophosphorylation in mammalian cell lines, including *N*-acetylglucosamine kinase (NAGK). Here, a detailed functional analysis of NAGK phosphorylation and pyrophosphorylation on serine 76 (S76) has been conducted. This analysis was enabled by using amber codon suppression to obtain phosphorylated pS76-NAGK, which was subsequently converted to site-specifically pyrophosphorylated NAGK (ppS76-NAGK) with a phosphorimidazolide reagent. A significant reduction in GlcNAc kinase activity was observed upon phosphorylation and near-complete inactivation upon pyrophosphorylation. The formation of ppS76-NAGK proceeded via an ATP-dependent autocatalytic process, and once formed, ppS76-NAGK displayed notable stability toward dephosphorylation in mammalian cell lysates. Proteomic examination unveiled a distinct set of protein–protein interactions for ppS76-NAGK, suggesting an alternative function, independent of its kinase activity. Overall, a significant regulatory role of pyrophosphorylation on NAGK activity was uncovered, providing a strong incentive to investigate the influence of this unusual phosphorylation mode on other kinases.

## Introduction

The covalent post-translational modification (PTM) of proteins is a widely used mechanism to regulate protein structure and function within cells, and an impressive array of PTMs has been documented to date.^1^ Protein phosphorylation is one of the most prevalent modifications and plays a central role in cellular signal transduction processes. It has been estimated that 30–65% of the human proteome are phosphorylated at some point in time, however, the functional relevance for the majority of phosphorylation sites remains unclear.^2,3^

To interrogate the impact of phosphorylation on protein structure and activity, access to stoichiometrically phosphorylated proteins is of great benefit. Chemical tools offer a precise means to synthesize stoichiometrically phosphorylated proteins. Protein semisynthesis, a combination of solid-phase peptide synthesis to include the desired phosphorylation site and protein expression of protein fragments, is a powerful technique to obtain stoichiometrically modified proteins and has been widely applied.^4−7^ In addition, the stoichiometric and site-specific incorporation of unnatural amino acids via genetic code expansion has recently been advanced to include the incorporation of phosphoserine, phosphothreonine, and phosphotyrosine.^8−11^

Adding to the complexity of phosphorylation-based signaling, a novel, nonenzymatic modification, termed protein pyrophosphorylation, was reported in 2007.^12^ This modification is thought to be mediated by inositol pyrophosphate messengers (PP-InsPs), which putatively transfer their β-phosphoryl group to prephosphorylated protein substrates, resulting in the formation of a diphosphate (pyrophosphate) moiety.^12,13^ Our group recently developed a mass spectrometry approach to identify many endogenous mammalian pyrophosphorylation sites within complex samples.^14^ The pyrophosphorylation sites were typically detected on nuclear and nucleolar proteins, and the sites localized to acidic polyserine stretches with a high degree of disorder.

**Figure 1:**
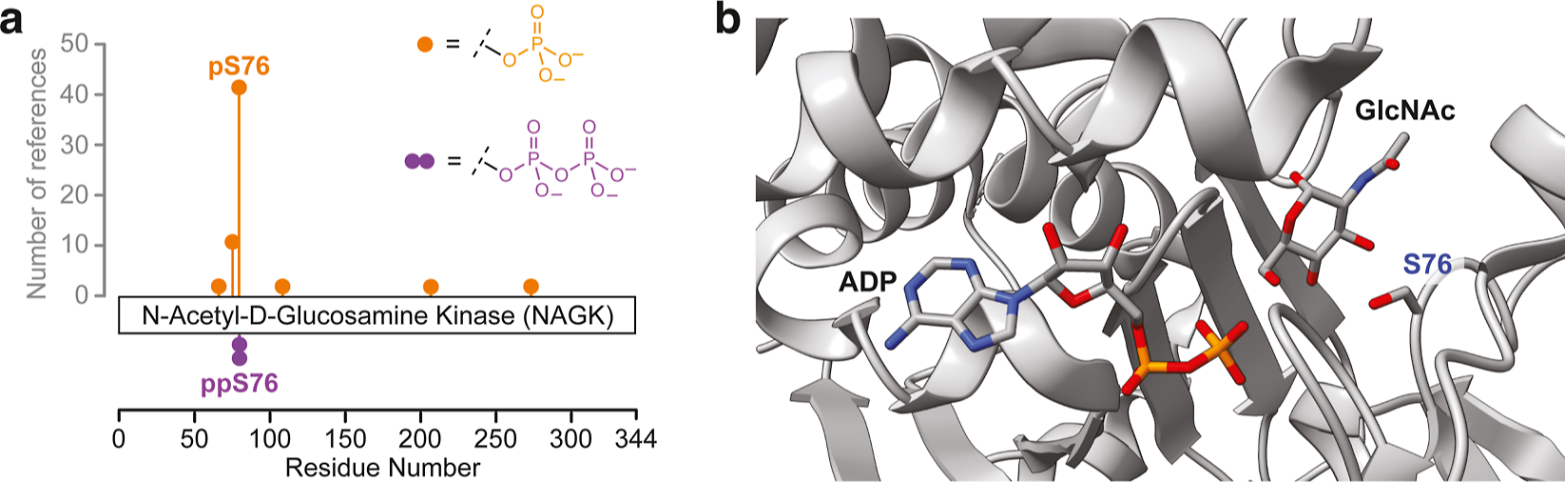
(a) Reported phosphorylation sites on NAGK in PhosphoSitePlus^25^ and previously discovered pyrophosphorylation site ppS76 reported by Morgan et al.^14^ (b) Structures of NAGK cocrystallized with ADP and GlcNAc, highlighting the location of S76.^15^ Two structures (PDB codes 2CH5 and 2CH6) were overlaid. Carbon atoms are shown in gray, oxygen atoms in red, nitrogen atoms in blue, and phosphorus atoms in orange.

However, a few pyrophosphoproteins did not fit that picture, among them *N*-acetylglucosamine kinase (NAGK, Figure 1a). In NAGK, the pyrophosphorylation site is in a structurally resolved area of the protein, immediately adjacent to the substrate binding site (Figure 1b). NAGK is best known for its participation in the *N*-acetylglucosamine (GlcNAc) salvage pathway, where it catalyzes the phosphorylation of GlcNAc to produce GlcNAc-6-phosphate.^15−17^ While GlcNAc-6-phosphate is typically synthesized de novo via the hexosamine biosynthesis pathway, in nutrient-deprived microenvironments cellular GlcNAc-6-phosphate concentrations can be maintained at a high level by an increased expression of NAGK and heightened GlcNAc salvage.^18^

In addition to its capacity to phosphorylate GlcNAc, NAGK can phosphorylate peptidoglycans, specifically muramyl dipeptide (MDP), to form 6-*O*-phospho-MDP in a process that triggers pro-inflammatory gene expression.^19,20^ Finally, a few reports have recently ascribed a scaffolding function to NAGK—independent from its catalytic activity.^21−24^

Considering the central role of NAGK in the GlcNAc salvage pathway and its additional functions, it is important to understand how NAGK activity and function are regulated. While several sites of modification—predominantly phosphorylation—of NAGK have been identified in high-throughput studies, the functional relevance of these modification sites has not been addressed.^25^ We were particularly interested in the phosphorylation site on serine 76 (S76) because it is the most commonly detected phosphorylation site on NAGK (Figure 1a). In addition, S76 was recently found to be pyrophosphorylated and is positioned in close proximity to the substrate binding pocket (Figure 1b).^14,15^

To elucidate the influence of phosphorylation and pyrophosphorylation at S76 on NAGK, we now report the synthesis of site-specifically phosphorylated and pyrophosphorylated NAGK. Access to these stoichiometrically modified protein samples enabled the detailed characterization of the different phosphorylation modes. Compared to the unmodified protein, the GlcNAc kinase activity of the phosphoprotein was significantly reduced. The pyrophosphoprotein was almost completely inactive. This inactivation appeared to be irreversible, as the pyrophosphoryl group on NAGK was resistant to hydrolysis in active cell lysates. Instead, pyrophosphorylation bestows NAGK with the ability to physically interact with a distinct set of proteins, compared to the unmodified protein. Overall, our findings highlight the intricacies of phospho-regulation, where a small change in protein modification can bring about large alterations in function and will motivate us and others to further explore the biochemical and biophysical properties of other pyrophosphorylated kinases.

## Results

### Synthesis of Pyrophosphorylated NAGK

**Figure 2:**
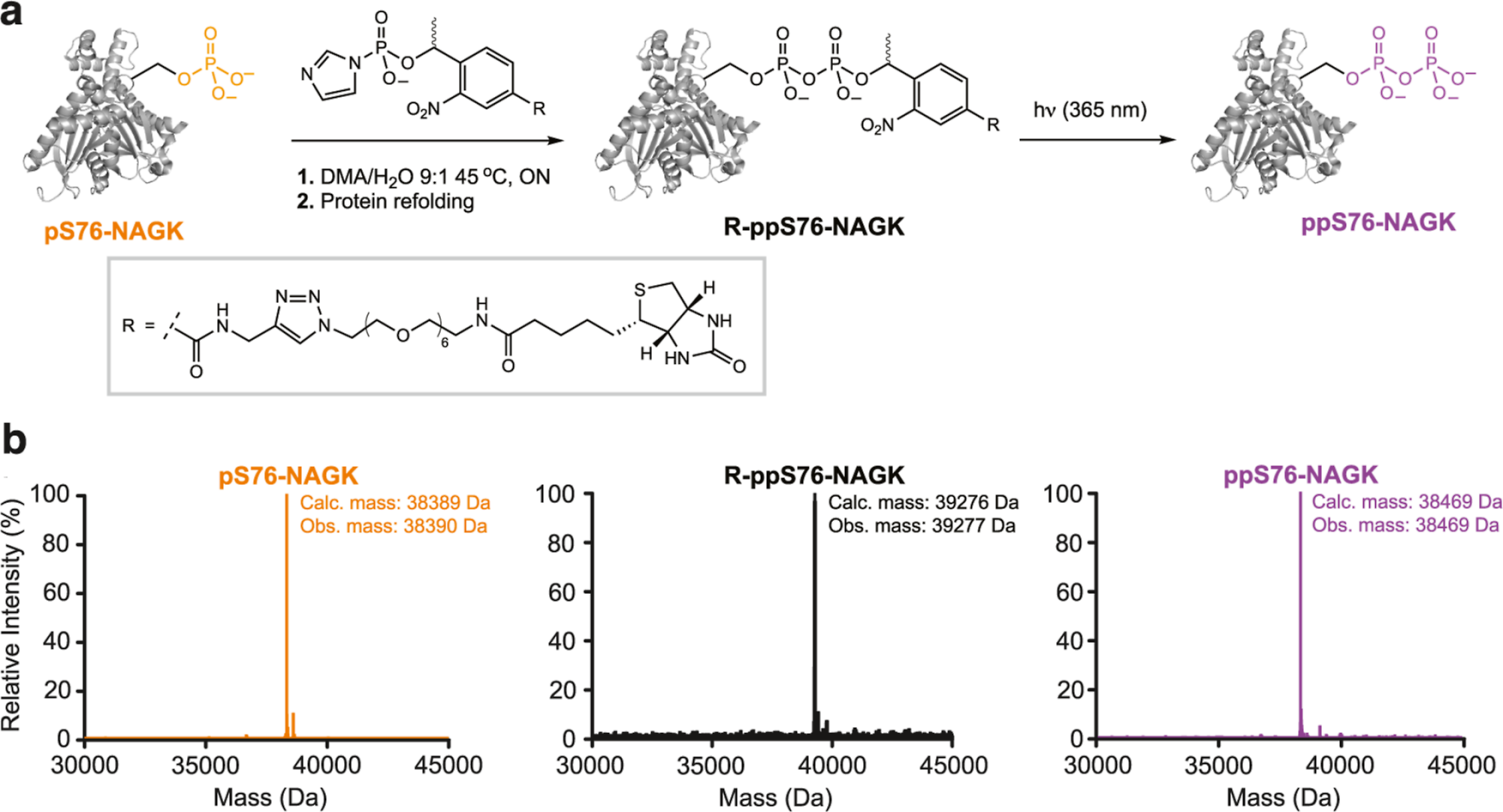
(a) Chemical phosphorylation of pS76-NAGK to provide pyrophosphoprotein ppS76-NAGK. pS76-NAGK is derivatized by using biotin-PEG_6_-Tz-NPE-P-imidazolide for the selective modification of the phosphoserine moiety to yield R-ppS76-NAGK. Subsequent irradiation releases the pyrophosphoprotein ppS76-NAGK. (b) Deconvoluted Q-TOF-MS spectra of the intermediates and products of the reaction sequence in (a) are shown.

To investigate how pyrophosphorylation on S76 may affect the properties and function of NAGK, site-specifically and stoichiometrically modified protein is required. Based on previous work, we sought to apply a combination of amber codon suppression to incorporate phosphoserine, followed by a chemoselective reaction of the phosphoprotein with a photo-labile phosphorimidazolide (P-imidazolide) reagent, to obtain the corresponding pyrophosphoprotein.^26,27^ Expression of the phosphoprotein in *Escherichia coli* proceeded smoothly and yielded good quantities of pS76-NAGK (7.5 mg/L) in high purity (Figures S1 and S2). pS76-NAGK was subsequently treated with biotin-polyethylenglycol-6-triazole-nitrophenylethyl-phosphorimidazolide (biotin-PEG_6_-Tz-NPE-P-imidazolide) for 18 h at 45 °C in a solvent mixture of DMA and H_2_O (Figure 2a). After refolding, the formation of the derivatized protein, R-ppS76-NAGK, was clearly observed by quadrupole time-of-flight mass spectrometry (Q-TOF-MS, Figure 2b). To release the pyrophosphoprotein, the sample was exposed to 365 nm light, and ppS76-NAGK could be isolated without notable formation of any side products (Figure 2b).

To confirm that the refolding step was suitable to obtain properly folded protein, we subjected wildtype-NAGK (wt-NAGK) to the derivatization/refolding conditions. Circular dichroism measurements corroborated the successful restoration of protein structure (Figures S3 and S4), and kinase activity was unaltered. In sum, the site-specifically modified phosphoprotein pS76-NAGK and pyrophosphoprotein ppS76-NAG could be accessed readily, alongside the unmodified protein, wt-NAGK.

### Pyrophosphorylation Severely Reduces GlcNAc Kinase Activity

**Figure 3:**
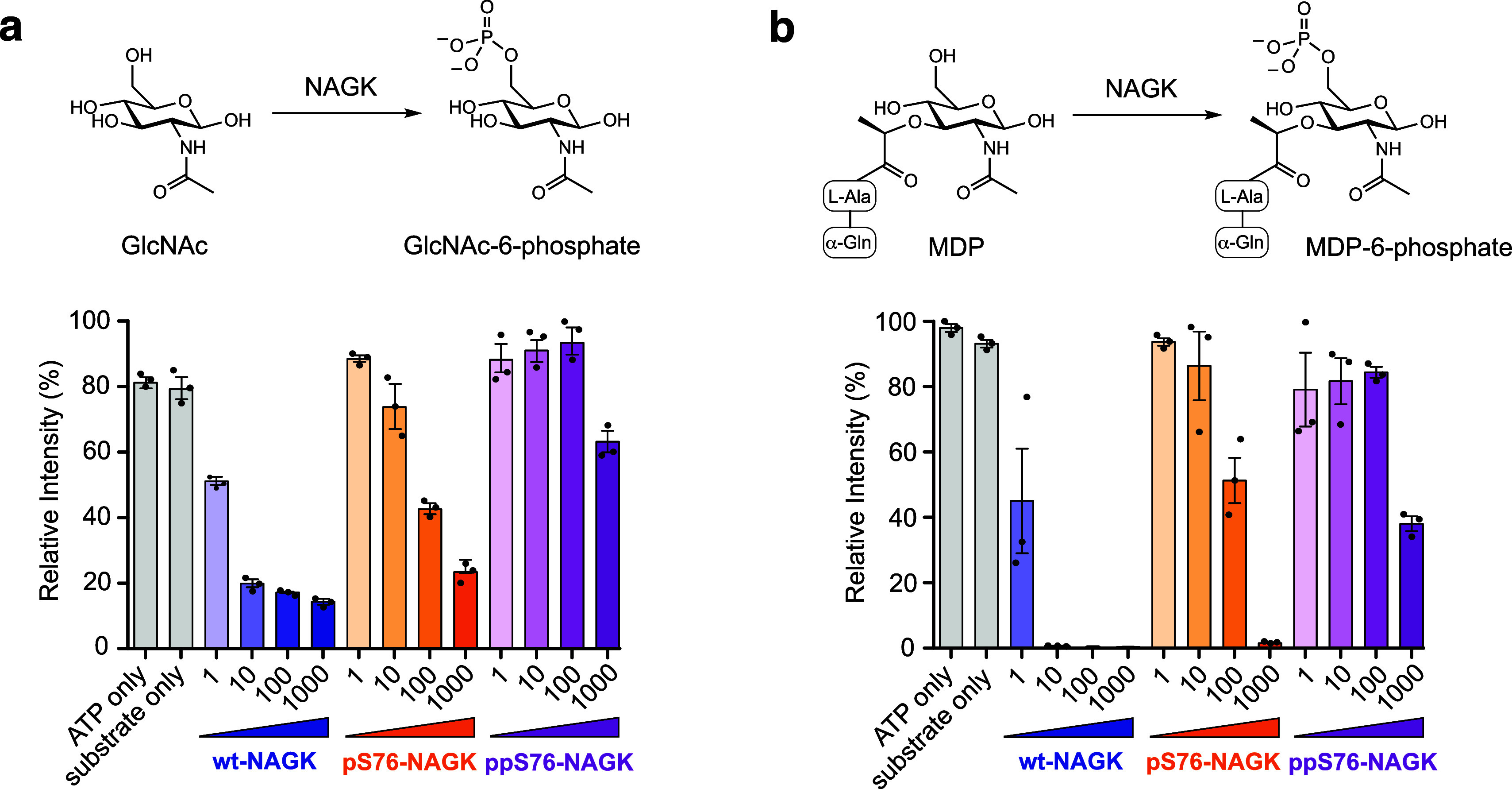
Phosphorylation and pyrophosphorylation of NAGK reduce the enzymatic activity. (a) NAGK kinase activity utilizing GlcNAc as a substrate was measured at 37 °C for 1 h in 50 mM HEPES (pH 7.5), 50 mM NaCl, 10 mM MgCl_2_, 70 μM GlcNAc, and 100 μM ATP. Concentrations of NAGK (1–1000) are in nM. (b) NAGK kinase activity utilizing MDP as a substrate was measured at 37 °C for 1 h in 50 mM HEPES (pH 7.5), 50 mM NaCl, 10 mM MgCl_2_, 190 μM MDP, and 100 μM ATP. Concentrations of NAGK (1–1000) are in nM. Data is presented as mean ± standard error (SE) of three technical replicates.

With wt-NAGK, pS76-NAGK, and ppS76-NAGK in hand, we next wanted to evaluate how the different phosphorylation modes influenced the enzymatic activity of NAGK. To do so, a standard kinase assay was set up using GlcNAc as a substrate and monitoring adenosine-5′-triphosphate (ATP) consumption (Figure 3a).^28^ At low enzyme concentration (1 nM), unphosphorylated wt-NAGK displayed a robust activity of 260 nmol/min/ng. By contrast, pSer76-NAGK showed no activity at 1 nM enzyme concentration. Even with increased enzyme concentration, its overall activity was decreased by approximately 200-fold to 1.4 nmol/min/ng (Figures 3a, S5). Pyrophosphorylation on S76 decreased the kinase activity even more, and low conversion only became detectable at an enzyme concentration of 1 μM (Figure 3a).

In addition to its GlcNAc kinase activity, it was recently shown that NAGK can phosphorylate MDP to generate MDP-6-phosphate in response to bacterial infection.^19^ We therefore tested the ability of wt-NAGK, pS76-NAGK, and ppS76-NAGK to utilize MDP as a substrate. Again, wt-NAGK exhibited high activity toward MDP, while the activity of pS76-NAGK was significantly reduced, and ppS76-NAGK showed even lower conversion (Figure 3b). Overall, the kinase activity of NAGK toward GlcNAc and MDP was strongly reduced by the introduction of a single phosphoryl group on S76. Pyrophosphorylation on this side chain caused an even more pronounced effect, making NAGK virtually inactive.

### Phosphorylation by Aurora Kinase B Is Followed by Autopyrophosphorylation

**Figure 4:**
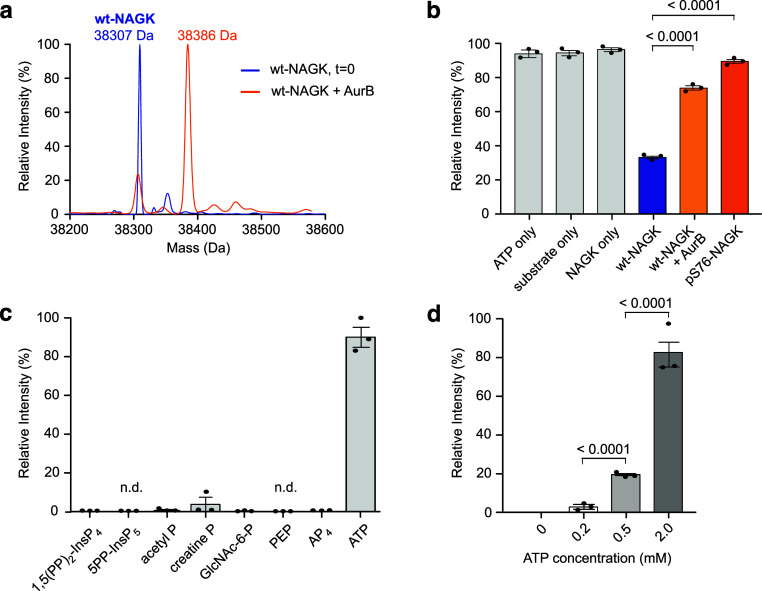
Phosphorylation and pyrophosphorylation of NAGK. (a) AurB phosphorylates NAGK. Deconvoluted Q-TOF-MS spectra of wt-NAGK before (blue) and after (orange) incubation with AurB are shown. (b) Measurement of GlcNAc kinase activity of wt-NAGK, before and after treatment with AurB. The activity of pS76-NAGK is shown for comparison. (c) Investigation of the ability of different metabolites to serve as phosphoryl donors in potential pyrophosphorylation reactions. Assays were performed by incubating 10 μM pS76-NAGK and 0.2 mM phosphoryl donor in 50 mM HEPES (pH 7.5), 150 mM NaCl, 0.8 mM MgCl_2_, and 1 mM DTT at 37 °C overnight. Data is presented as mean ± SE of three technical replicates. (d) Relative quantification of pyrophosphorylation at different ATP concentrations. Assays were performed by incubating 10 μM pS76-NAGK and 0–2 mM ATP in 50 mM HEPES (pH 7.5), 150 mM NaCl, 0–2 mM MgCl_2_, and 1 mM DTT at 37 °C overnight. Data is presented as mean ± SE of three technical replicates. P-values were determined by unpaired *t*-test analysis.

Considering the pronounced effect of a single phosphorylation/pyrophosphorylation event on NAGK activity, we wondered how these modifications were installed. To date, no biochemical data on protein kinases targeting S76 on NAGK have been reported. Nonetheless, a prior high-throughput proteomic investigation identified this residue as a candidate site for phosphorylation by aurora kinase B (AurB) and cyclin-dependent kinase 1 (CDK1).^29,30^ To validate this potential connection, AurB was incubated with wt-NAGK in the presence of ATP and MgCl_2_. Q-TOF-MS corroborated the addition of the phosphoryl group to NAGK (Figure 4a), and MS/MS analysis confirmed the addition to serine 76 (Figure S6).^31^ Phosphorylation by AurB notably reduced GlcNAc kinase activity, consistent with our previous observation using recombinantly expressed pS76-NAGK (Figure 4b). In addition, CDK1 also proved to be a competent protein kinase for NAGK phosphorylation (Figure S7).

Protein pyrophosphorylation is a recently emerging modification and is thought to be mediated nonenzymatically by inositol pyrophosphate metabolites (PP-InsPs).^12,13,32−36^ Therefore, we tested whether ppS76-NAGK would form upon incubation of pS76-NAGK with PP-InsPs, specifically, 5-diphosphoinositol pentakisphosphate (5PP-InsP_5_) and 1,5-bisdiphosphoinositol tetrakisphosphate [1,5(PP)_2_-InsP_4_] in the presence of Mg^2+^ ions. Interestingly, neither 5PP-InsP_5_ nor 1,5(PP)_2_-InsP_4_ were capable of generating the pyrophosphoprotein, even after extended reaction times (Figure 4c). While the product of the 1,5(PP)_2_-InsP_4_ reaction indicated signs of the mass corresponding to pyrophosphorylation at the MS1 level, MS/MS analysis did not confirm product formation. Because mammalian cells contain various high-energy metabolites that could serve as phosphoryl donors, we subsequently investigated the ability of ATP, adenosine-5′-tetraphosphate (AP_4_), phosphoenolpyruvate (PEP), GlcNAc-6-phosphate, creatine phosphate, and acetyl phosphate to participate in phosphoryl transfer chemistry. For acetyl phosphate, creatine phosphate, GlcNAc-6-phosphate, PEP, and AP_4_, again, the formation of ppS76-NAGK was not detected by MS/MS (Figure 4c). However, upon treatment of pS76-NAGK with 200 μM ATP, a robust signal for the corresponding pyrophosphopeptide became apparent in the MS1 spectrum, and the pyrophosphoryl group could be localized by MS/MS analysis (Figure S8).^31^ Since no additional enzymes were present in these biochemical reactions, the generation of the pyrophosphoryl moiety appears to be an autocatalytic event. The degree of autopyrophosphorylation should therefore correlate with the ATP concentration. Indeed, the signal for the pyrophosphopeptide increased when ATP concentrations were elevated (Figure 4d). Overall, the formation of the pyrophosphate group on ppS76-NAGK is not mediated by PP-InsPs but is generated in an ATP-dependent, autocatalytic fashion. Considering the close proximity between the ATP binding site and pS76 (Figure 1b), an intramolecular phosphoryl transfer reaction appears feasible. Given the unusual reaction sequence that leads to pyrophosphorylation, the question arises if, and how, this modification can be removed.

### ppS76-NAGK Is Resistant to Dephosphorylation in Mammalian Cell Lysates

**Figure 5:**
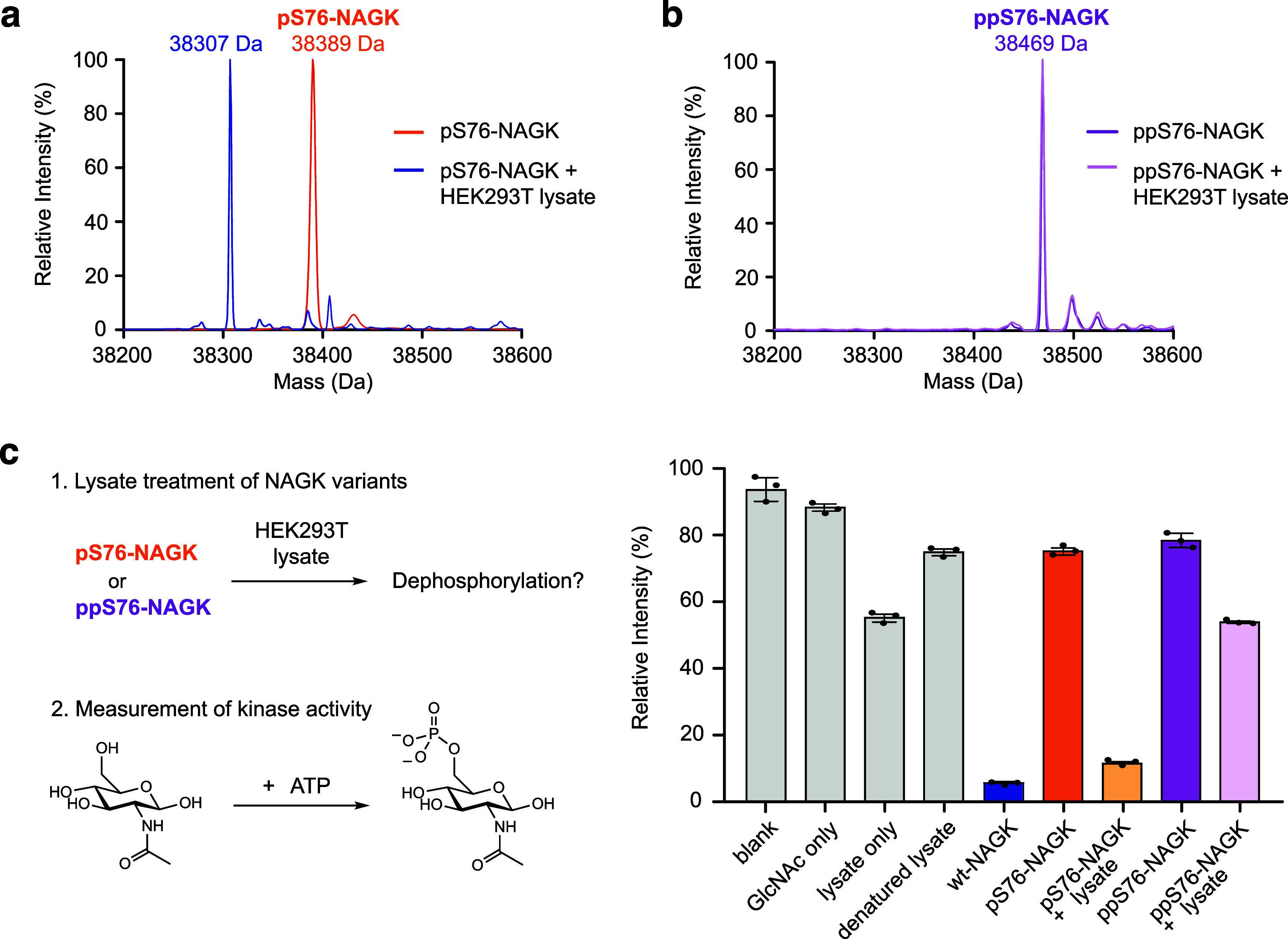
Dephosphorylation of pS76-NAGK and ppS76-NAGK. (a) pS76-NAGK (orange) followed by treatment with HEK293T cell lysate (blue), indicating dephosphorylation. Deconvoluted Q-TOF-MS spectra are shown. (b) ppS76-NAGK (purple) followed by treatment with HEK293T cell lysates (light purple) is shown which indicates no conversion. Deconvoluted Q-TOF-MS spectra are shown. (c) Assessment of potential (pyro)phosphatases in mammalian cell lysates. Biochemical determination of the activity of pS76-NAGK or ppS76-NAGK following treatment with HEK293T lysate. Experiments with HCT116 and PANC-1 cell lysates displayed similar results (Figure S8) Data is presented as mean ± SE of three technical replicates. The bar graph for (c) including the *t*-test is shown in Figure S8.

To maintain dynamic control over protein function, phosphorylation of proteins is typically reversible and dephosphorylation is catalyzed by protein phosphatases.^37−39^ Phosphatases that would remove one or two phosphoryl groups from pyrophosphoproteins have not been described to date. To investigate possible dephosphorylation reactions, we prepared cell lysates from HEK293T cells and first monitored the dephosphorylation of pS76-NAGK by Q-TOF-MS (Figure 5a). High conversion of pS76-NAGK to the dephosphorylated product was observed, validating the activity of the lysate. In contrast, ppS76-NAGK displayed no hydrolysis at all, even after extended incubation times, and used different Lewis acidic metal cations in the buffer, demonstrating a high degree of biochemical stability of the pyrophosphoserine group (Figure 5b). The inertness of ppS76-NAGK toward hydrolysis was also maintained in lysates from human colon colorectal carcinoma cells (HCT116) and human pancreas-1 (PANC-1) cells (Figure S9).

Since dephosphorylation of NAGK should restore its GlcNAc kinase activity, we next probed this activity upon incubation of pS76-NAGK and ppS76-NAGK with cell lysate. As expected, pS76-NAGK regained kinase activity upon exposure to HEK293T lysates, whereas ppS76-NAGK did not (Figure 5c). The residual kinase activity observed in the ppS76-NAGK sample can be attributed to the presence of ATPases in the lysate, leading to ATP consumption (Figure 5c). These results are in line with previous work using radiolabeled pyrophosphoproteins, where a resistance toward dephosphorylation by common protein phosphatases was reported.^13^ The increased stability of the pyrophosphorylation mark (compared to monophosphorylation) seems to equip the proteins with a more permanent modification and may potentially play a role in mediating protein–protein interactions.^33,40^

### Pyrophosphorylation Influences the Protein–Protein Interactions of NAGK

A few recent examples have shown how protein pyrophosphorylation can either enhance or decrease the affinity of specific protein–protein interactions.^32−36,40^ To investigate how the protein interactome of ppS76-NAGK compared to wt-NAGK, lysates were prepared from HEK293T cells and were incubated with His_6_-tagged wt-NAGK or ppS76-NAGK. Following immobilization on nickel beads, the supernatants were removed, the protein complexes were washed, and subsequently eluted with imidazole.^41^ Analysis of the eluate by high-resolution mass spectrometry identified many known interactors of NAGK (Figure S10).^42−45^

**Figure 6:**
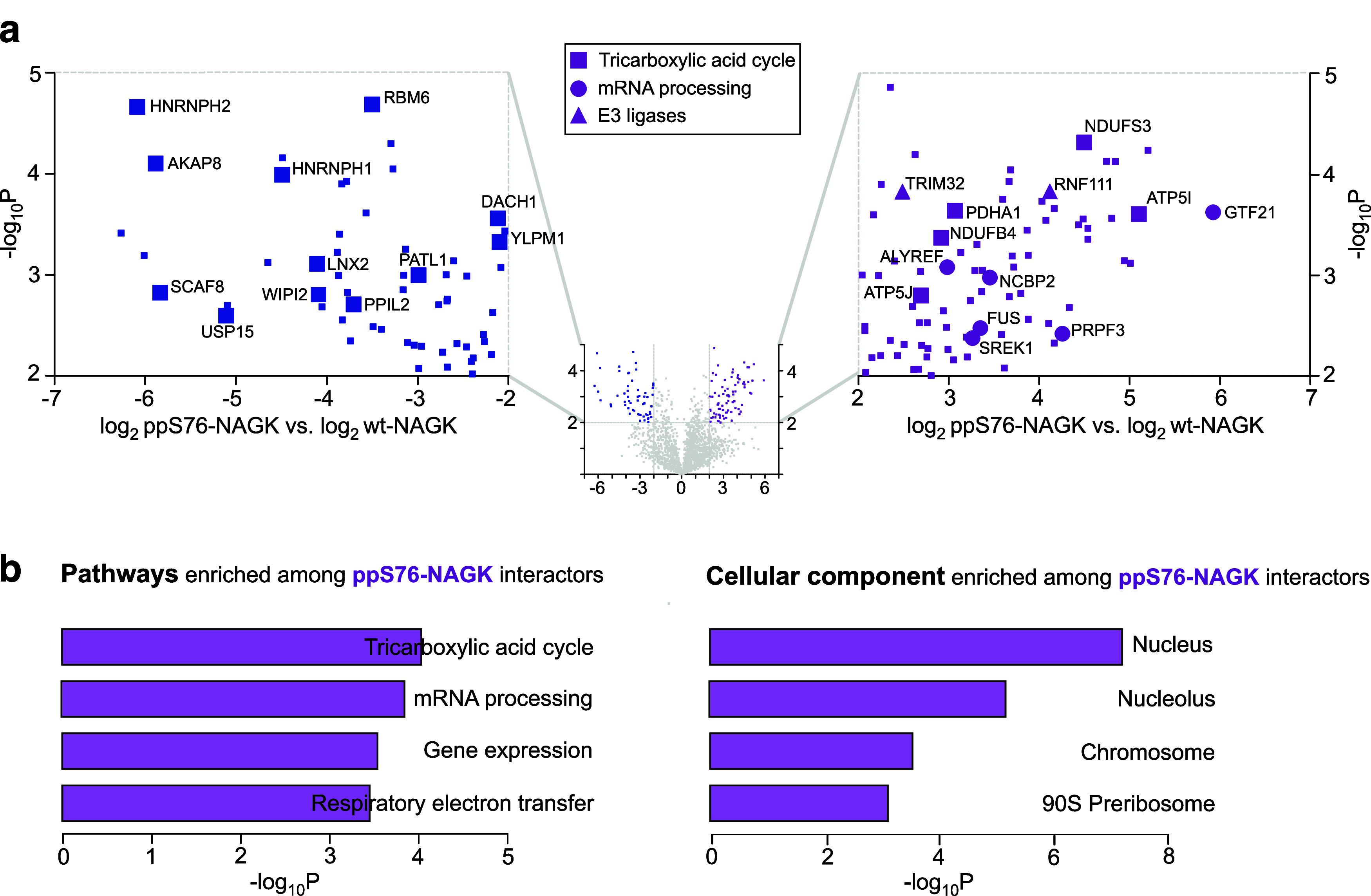
Interactome analysis of ppS76-NAGK versus wt-NAGK. (a) Volcano plot depicting LFQ values of ppS76-NAGK versus wt-NAGK after a *t*-test. The *x*-axis displays the difference of LFQ values on a log_2_ scale and the *y*-axis shows the −log_10_*P* value. The left side (blue) illustrates the preferential enrichment with wt-NAGK and includes several known binding partners (blue large squares). The right side illustrates the preferential enrichment with ppS76-NAGK (purple). Examples of over represented pathways are highlighted. (b) GO analysis of proteins that preferentially interact with ppS76-NAGK.

Next, a volcano plot was generated comparing LFQ values for ppS76-NAGK versus wt-NAGK to examine how protein–protein interactions were altered by pyrophosphorylation. Interestingly, several known NAGK interactors (AKAP8, HNRNPH1, HNRNPH2, SCAF8, RBM6, USP15, LNX2, WIPI2, PPIL2, PATL1, DACH1, and YLPM1) are no longer associated with pyrophosphorylated NAGK, suggesting that pyrophosphorylation destabilized these binding events (Figure 6a). On the contrary, not a single known NAGK interactor is preferentially bound to ppS76-NAGK. Instead, pyrophosphorylation appears to promote a distinct set of protein–protein interactions: 78 proteins were identified that preferentially interacted with ppS76-NAGK, either directly or indirectly (thresholds were set to log_2_ > 2 and -log_10_*P* > 2) (Figure 6a, Table S1).

To associate this list of 78 proteins with functional terms, we used Enrichr for gene ontology (GO) analysis (Table S2).^46,47^ This analysis suggests a role for ppS76-NAGK in the tricarboxylic acid (TCA) cycle and respiratory electron transport, as proteins from these pathways (ATP5I, ATP5J, PDHA1, NDUFB4, and NDUFS3) are overrepresented among the ppS76-NAGK interactors (Figure 6b). Additionally, ppS76-NAGK binds to several proteins involved in mRNA processing (GTF2F1, FUS, SREK1, NCBP2, and PRPF3). Consistent with the association with mRNA processing, the nucleus is the cellular component most strongly enriched among ppS76-NAGK interactors (Figure 6b). Finally, we identified two E3 ligases, TRIM32 and RNF111, that preferentially interacted with ppS76-NAGK. Given the biochemical stability of pyrophosphorylation, it seems plausible that—instead of dephosphorylation—ubiquitin-mediated degradation may be involved in the turnover of pyrophosphorylated NAGK.^40^

## Discussion

In this study, we could discern the influence of pyrophosphorylation and phosphorylation on NAGK activity, which was facilitated by the ability to obtain stoichiometrically and site-specifically modified protein. We first expressed pS76-NAGK, using amber codon suppression. Application of chemoselective P-imidazolide chemistry led to the successful generation of ppS76-NAGK in good yield. In principle, this approach should be readily extendable to other proteins of interest, in which these different phosphorylation modes have been observed. However, the current method to generate pyrophosphoprotein requires the use of organic solvent to ensure full conversion and precise protein modification. While the refolding of ppS76-NAGK proceeded smoothly, refolding conditions likely have to be adjusted and optimized for other proteins. It would therefore be desirable to develop P-imidazolide reagents that can operate in aqueous environments to preserve protein structure during the course of the reaction.^48^

**Figure 7:**
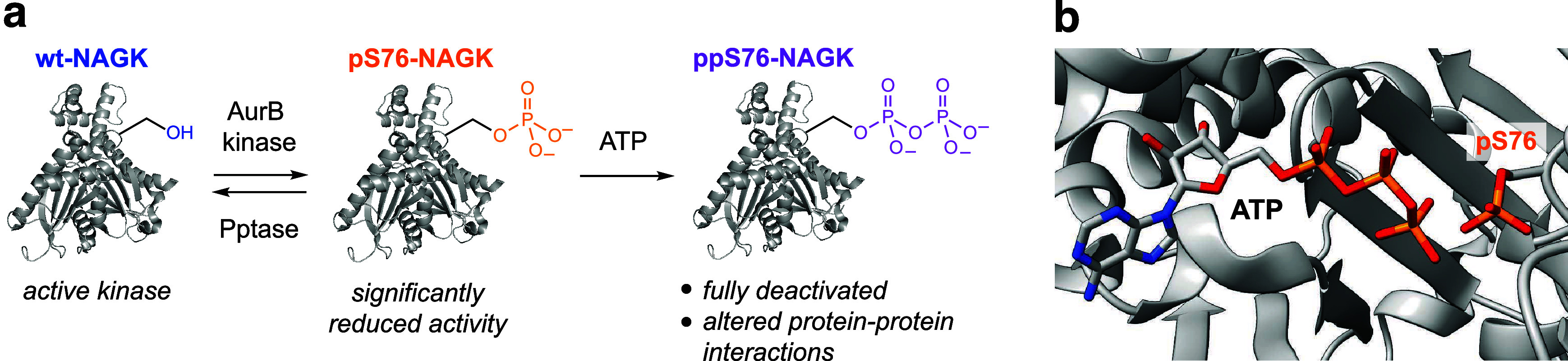
(a) Summary of the impact of phosphorylation and pyrophosphorylation on the function of NAGK. Pptase is short for phosphatase. (b) Structural model of ATP bound to pS76-NAGK, based on the NAGK-ADP structure (PDB: 2CH6).

When pS76-NAGK and ppS76-NAGK were assayed for their kinase activity, a stepwise decrease was observed, compared to wt-NAGK: phosphorylation led to a significant reduction in activity, while pyrophosphorylation resulted in almost complete deactivation (Figure 7a). The available crystal structure of wt-NAGK illustrates the close proximity between the ATP-binding site and S76.^15^ Phosphorylation at this site would lead to electrostatic repulsion between the modification and the triphosphate moiety of ATP. This repulsion would decrease ATP-binding and thereby slow down the transfer of the γ-phosphoryl group onto GlcNAc (or MDP). Consistent with this hypothesis, the effect of pyrophosphorylation should be even more pronounced, as was indeed observed.

The proximity of the ATP-binding site to pS76 also helps to understand the autocatalytic mechanism, which is proposed for the installation of the pyrophosphate group. While we initially suspected that PP-InsPs would serve as phosphoryl donors for the generation of ppS76-NAGK, these molecules proved to be unreactive. Instead, ATP was the only high-energy phosphoryl donor that promoted the formation of ppS76-NAGK, and the conversion was proportional to ATP concentration. Inspection of the crystal structure of wt-NAGK, into which we modeled a phosphoserine side chain at S76, illustrates how the γ-phosphoryl group of ATP and pS76 are within 3.4 Å of each other, suggesting that an intramolecular phosphoryl transfer is well possible (Figure 7b).^49^

The initial phosphorylation on S76 can be catalyzed by the protein kinase AurB. Pyrophosphorylation thereafter is facilitated by the presence of ATP. Interestingly, autopyrophosphorylation appears to be strongly dependent on temperature and proceeds much faster at 37 °C, compared to 18 °C (Figure S11).

Considering that ATP concentrations can change in response to nutrient availability and can also vary depending on subcellular location,^50−52^ we postulate that this sequential phosphorylation mechanism holds the potential for efficiently regulating the GlcNAc salvage pathway. Such a mechanism appears well-suited for nutrient-rich conditions and consequently high ATP concentrations, where reliance on the GlcNAc salvage pathway may be dispensable.^18^

Once the pyrophosphate group is attached to NAGK, it is quite stable toward chemical and biochemical hydrolysis. Consistent with previous observations on protein pyrophosphorylation, extended incubation with active cell lysates did not lead to dephosphorylation of ppS76-NAGK. The more “energetic” pyrophosphoprotein turns out to be much longer lived than its monophosphorylated counterpart. This resilience toward dephosphorylation extends the deactivation period of kinase activity and suggests that an alternate functional outcome may be associated with pyrophosphorylation.

Although limited information is available regarding the kinase-independent functions of NAGK, a few recent studies have demonstrated that NAGK can indeed serve as a scaffold by engaging in various protein–protein interactions.^21−24^ The protein-binding partners of catalytically inactive ppS76-NAGK that we identified differed starkly from the binding partners of the unmodified protein. None of the modification-specific interactors had been identified previously, presumably because pyrophosphorylated NAGK only comprises a small fraction of the total protein amount. Notably, ppS76-NAGK is associated with several proteins involved in the TCA cycle and mRNA processing. If ppS76-NAGK indeed localizes to mitochondria and has moonlighting functions to regulate cellular ATP production is something to be investigated in the future.

The resilience of ppS76-NAGK toward dephosphorylation raises the question under which conditions this modification is reversible.^53^ While the lysate conditions cannot capture the complex environment of a cell, the lysate conditions were sufficient to dephosphorylate pS76-NAGK. However, the dephosphorylation of ppS76-NAGK may require a specific phosphatase activity within a certain compartment, such as mitochondria or the nucleus. Alternatively, the turnover of ppS76-NAGK may depend on a ubiquitin-mediated degradation pathway. A recent study highlighted that pyrophosphorylation of MYC induces polyubiquitination by an E3 ligase, regulating cell survival through a novel “pyrophosphodegron”.^40^ This intriguing mechanistic hypothesis might be relevant to the turnover of ppS76-NAGK as well, as we could identify two E3 ligases that associated with the pyrophosphoprotein.

## Conclusions

In conclusion, this study expands our current understanding of protein pyrophosphorylation. While the change in NAGK structure for the three variants—serine, phosphoserine, and pyrophosphoserine—is likely small, the properties have been altered significantly. This granular analysis was only made possible by the synthetic access to stoichiometrically modified NAGK. Several other kinases, including dual specificity protein kinase CLK1, glycogen synthase kinase-3α/β, and nucleoside diphosphate kinase A/B, have recently been reported to carry a pyrophosphoserine or pyrophosphothreonine residue close to the ATP-binding site. It will therefore be of great interest to elucidate if some of the observations made here about protein pyrophosphorylation—the decrease of catalytic activity, ATP-dependent autopyrophosphorylation, and resistance to dephosphorylation—can be extended to these other candidates. We believe that the reported synthetic strategy, using a combination of amber codon suppression and P-imidazolide chemistry, together with the analytical tools established here, will greatly facilitate these investigations in the future.

## Data Availability

Methods and materials as well as supporting figures and tables are available in the Supporting Information. The mass spectrometry proteomics data have been deposited to the ProteomeXchange Consortium via the PRIDE^54^ partner repository with the data set identifier PXD049339.

## Supporting Information Available

Detailed description of materials and experimental methods, mass spectra, and supporting figures and tables (PDF)
LFQ values for wt-NAGK and ppS76-NAGK interactome analysis (XLSX)
GO analysis of ppS76-NAGK interactors (XLSX)
