# Supplementary material for: An Uncommon Phosphorylation Mode Regulates the Activity and Protein Interactions of N-Acetylglucosamine Kinase
Source: J Am Chem Soc. 2024 May 11;146(21):14807–15. doi: 10.1021/jacs.4c03069 (PMC11140747; doi:10.1021/jacs.4c03069)
Supplement: Supplementary file 1 — ja4c03069_si_001.pdf [file ja4c03069_si_001.pdf]

Supporting Information

An uncommon phosphorylation mode regulates the activity and protein-interactions of N-acetylglucosamine kinase

Arif Celik[1,2], Ida Beyer[1,2], Dorothea Fiedler[1,2*]

Affiliations

[1]: Leibniz-Forschungsinstitut für Molekulare Pharmakologie, Robert-Rössle-Straße 10, 13125 Berlin, Germany

[2]: Institut für Chemie, Humboldt-Universität zu Berlin, Brook-Taylor-Str. 2, 12489 Berlin, Germany

* Corresponding author

e-mail: fiedler@fmp-berlin.de

Table of Contents

1. Supporting Figures……………………………….…………………………………..1—10
2. General Information…………………….………..……………………………..11—12
   2.1 Cloning, site-directed mutagenesis, expression and purification of recombinant human NAGK……………………………………………………………………………..12—15
   2.2 General protocol for protein pyrophosphorylation…………………….………….16
   2.3 General Protocol for NAGK biochemical assays……………………..…………..17
   2.4 General information for the interactome analysis…………………………..…17—19
   2.5 Chemical Synthesis and characterization……………………………………….....19
3. Q-TOF-MS spectra……………………………………......…………………..20—23
4. References………………………………………………...……………………...…24

1. Supporting Figures

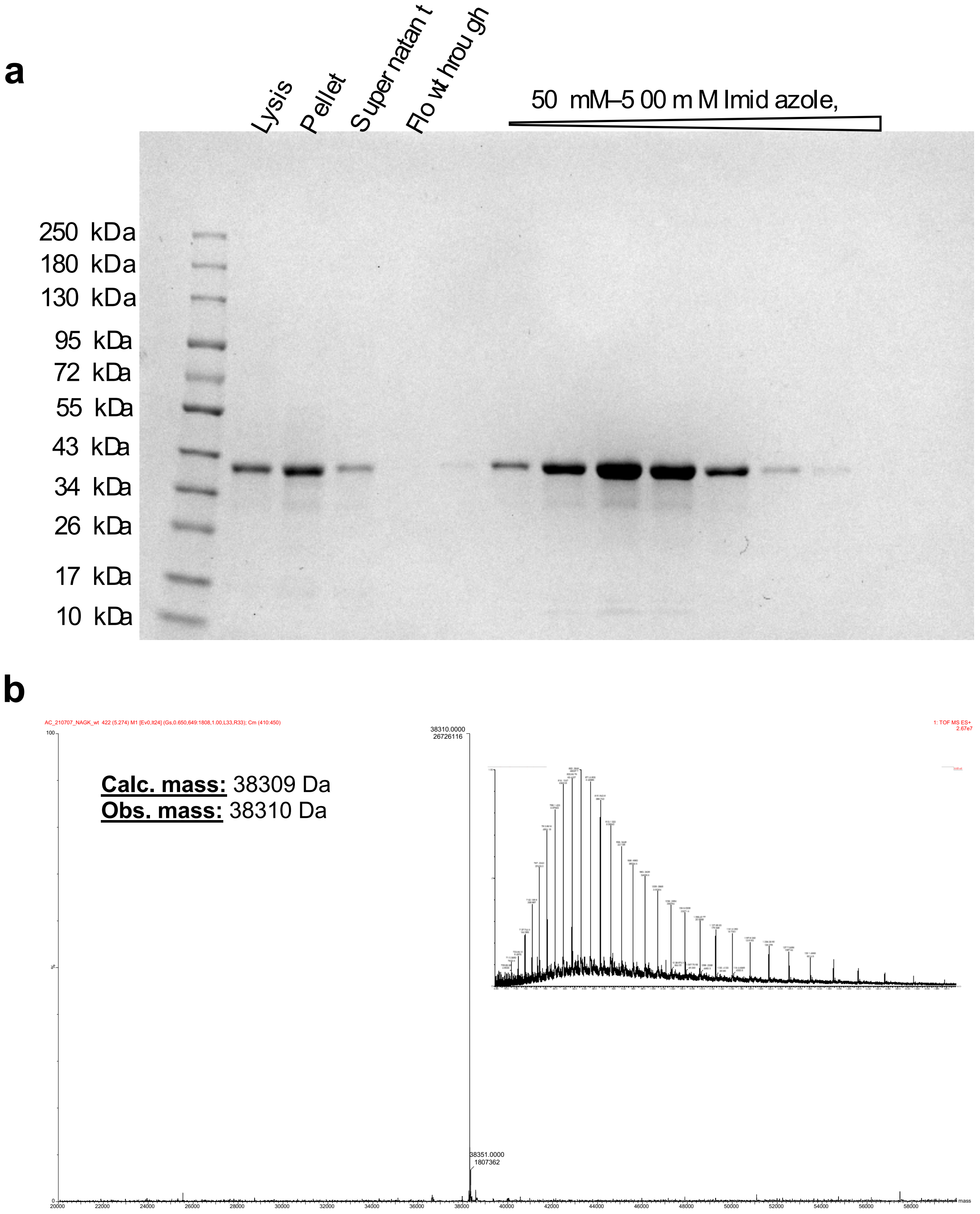

Figure S1. Recombinant expression of wt-NAGK. a) SDS-PAGE. b) Q-TOF-MS measurement.[1]

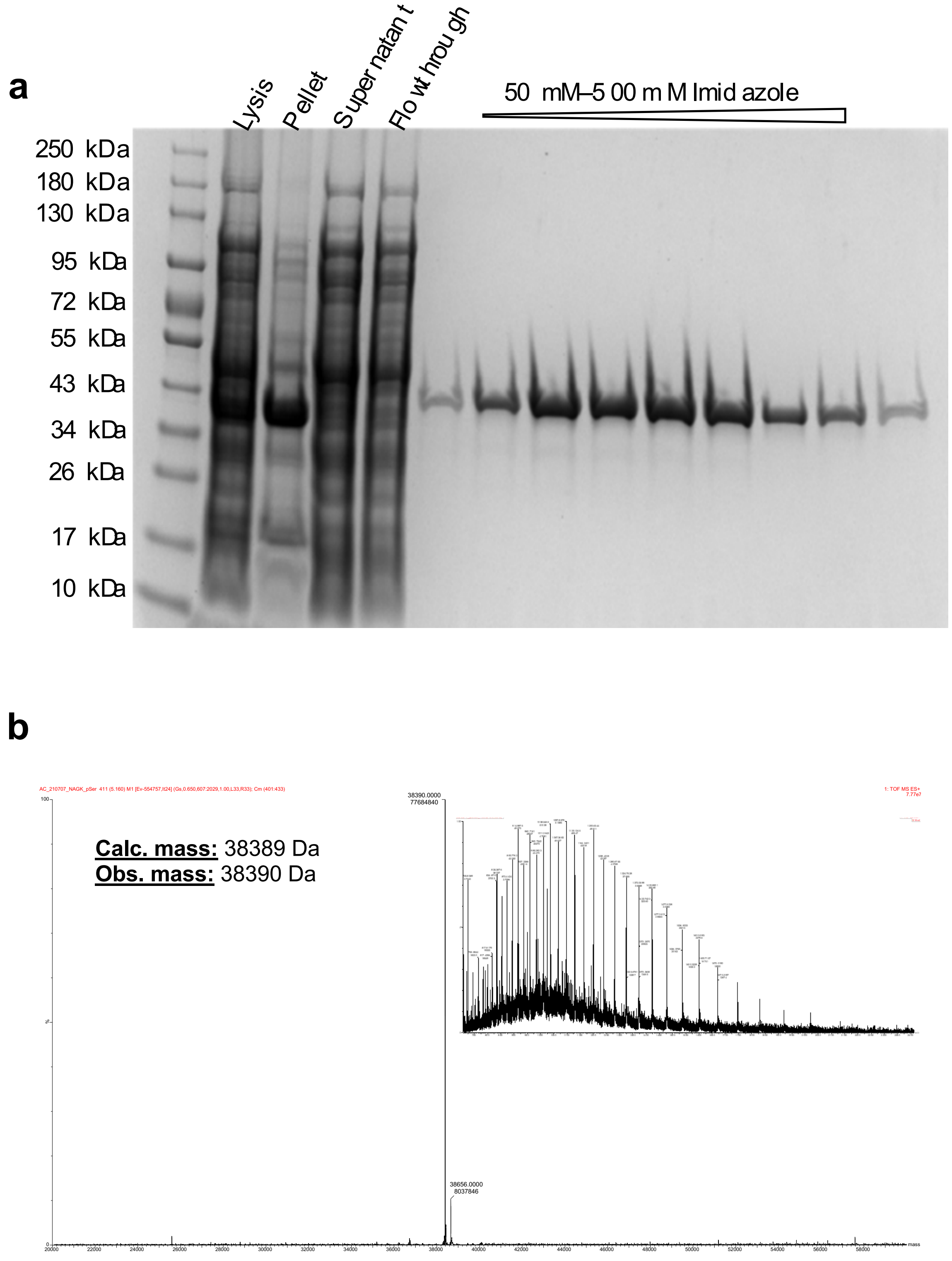

Figure S2. Recombinant expression of pS76-NAGK. a) SDS-PAGE b) Q-TOF-MS measurement.[1–2]

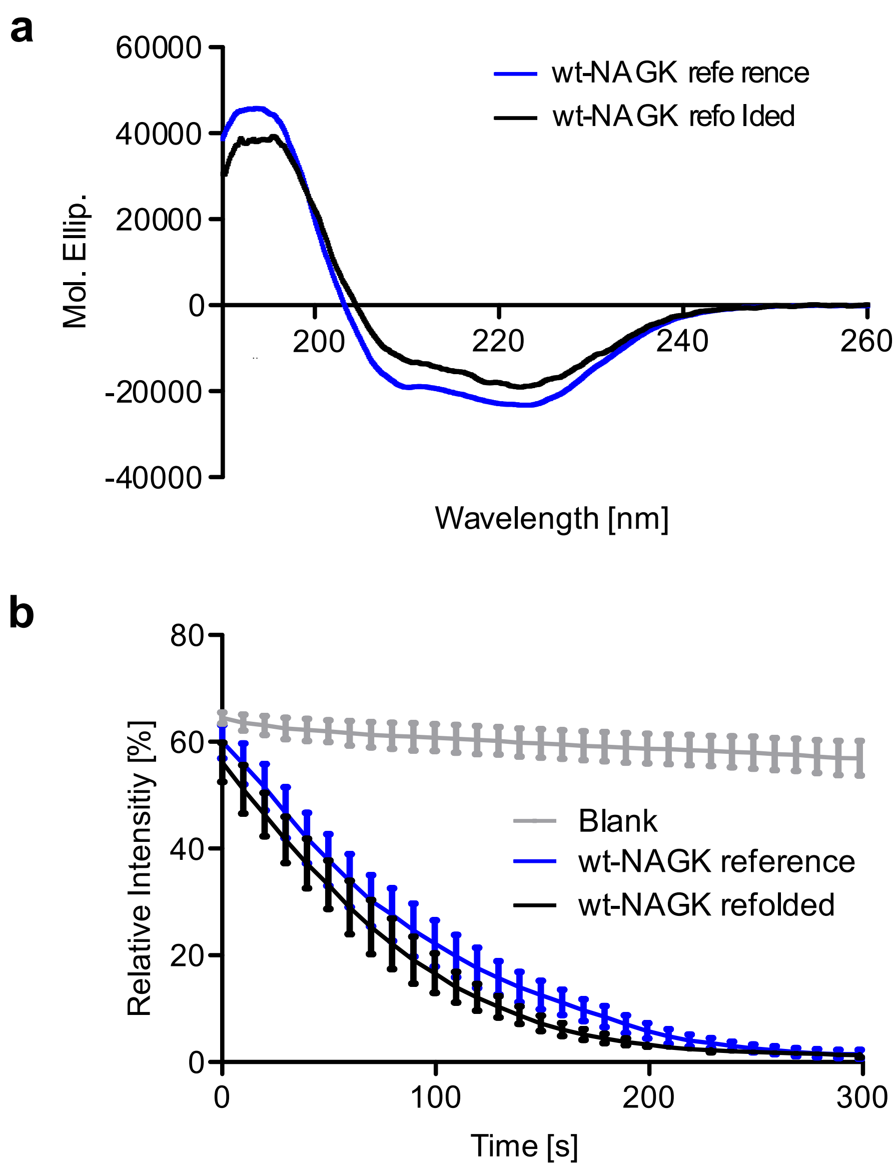

Figure S3. Assessment of NAGK secondary structure and activity before and after refolding. a) CD-Spectra of wt-NAGK before (in blue) and after refolding (in black). b) GlcNAc activity assay before and after refolding monitored by NADH consumption.

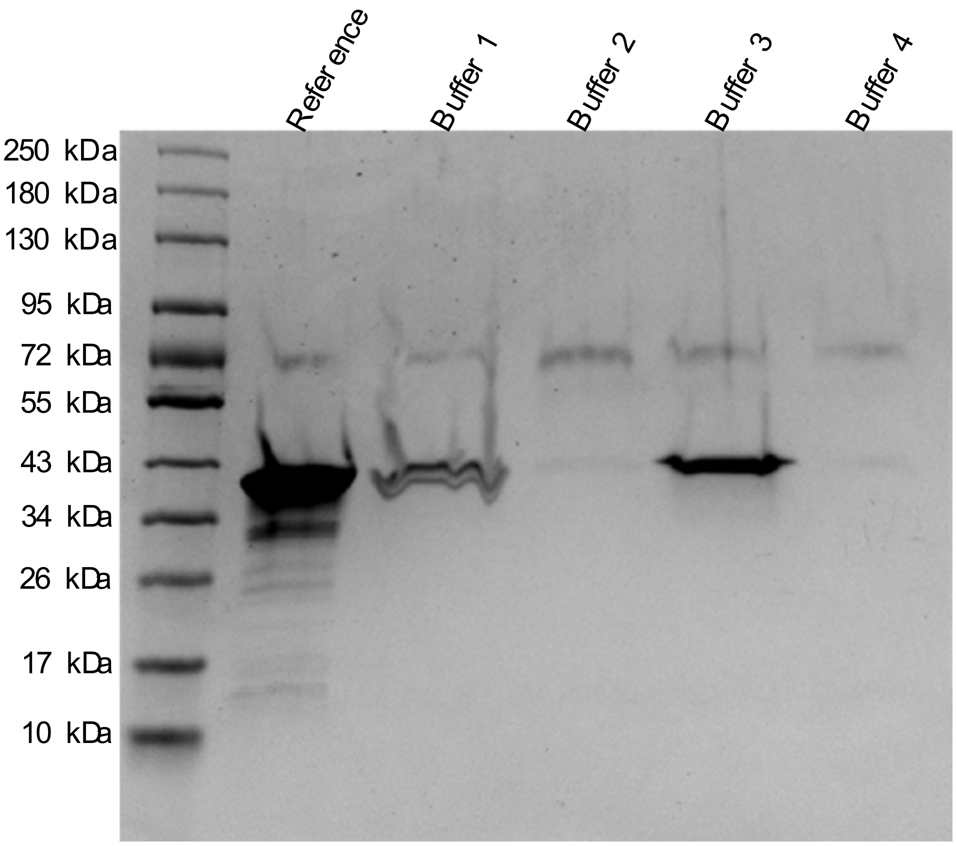

Figure S4. Refolding of wt-NAGK analyzed by SDS-PAGE. a) SDS PAGE after applying the refolding protocol on wt-NAGK. b) List of applied refolding buffers. **Buffer 1**: 50 mM Tris-HCl (pH 8.0), 250 mM NaCl, 20 mM DTT, 10 mM EDTA, 0.2% CHAPS, 1 mM GlcNAc. **Buffer 2**: 50 mM Tris-HCl (pH 8.0), 250 mM NaCl, 0.3 mM GSSG, 3 mM GSH, 10 mM EDTA, 0.2% CHAPS, 0.4 M Sucrose, 1 mM GlcNAc. **Buffer 3**: 50 mM Tris-HCl (pH 8.0), 250 mM NaCl, 0.3 mM GSSG, 3 mM GSH, 10 mM EDTA, 0.2% CHAPS, 0.1 M Arginine*HCl, 1 mM GlcNAc. **Buffer 4**: 50 mM Tris-HCl (pH 8.0), 250 mM NaCl, 20 mM DTT, 10 mM EDTA, 0.2% CHAPS, 5 mM GlcNAc, 10% Glycerol.[3,4]

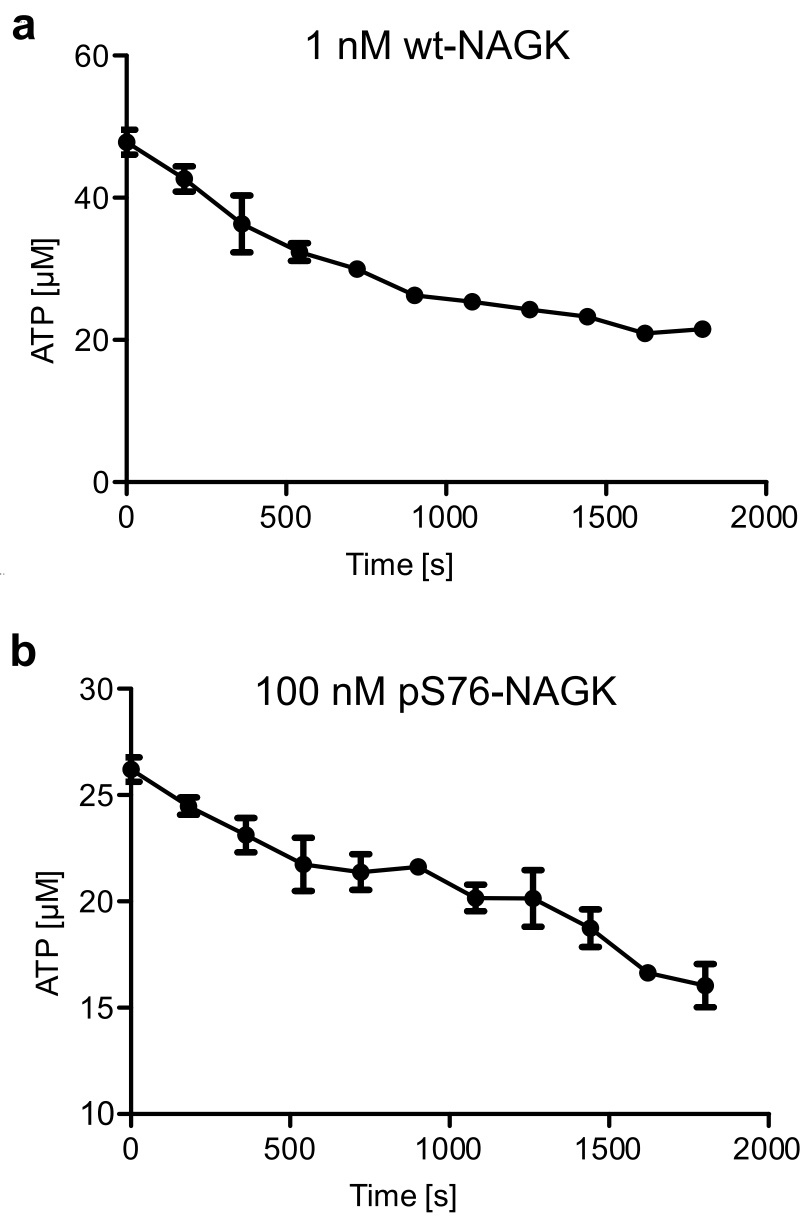

Figure S5. Determination of the activity of wt-NAGK and pS76-NAGK. ATP consumption was measured over time using *Promega* Kinase-Glo Plus® reagent, a) 1 nM wt-NAGK, 100 µM ATP, 50 mM HEPES, 100 mM NaCl, 10 mM $MgCl_2$, 1 mM DTT, and 70 µM GlcNAc. b) 100 nM pS76-NAGK, 100 µM ATP, 50 mM HEPES, 100 mM NaCl, 10 mM $MgCl_2$, 1 mM DTT, and 70 µM GlcNAc.

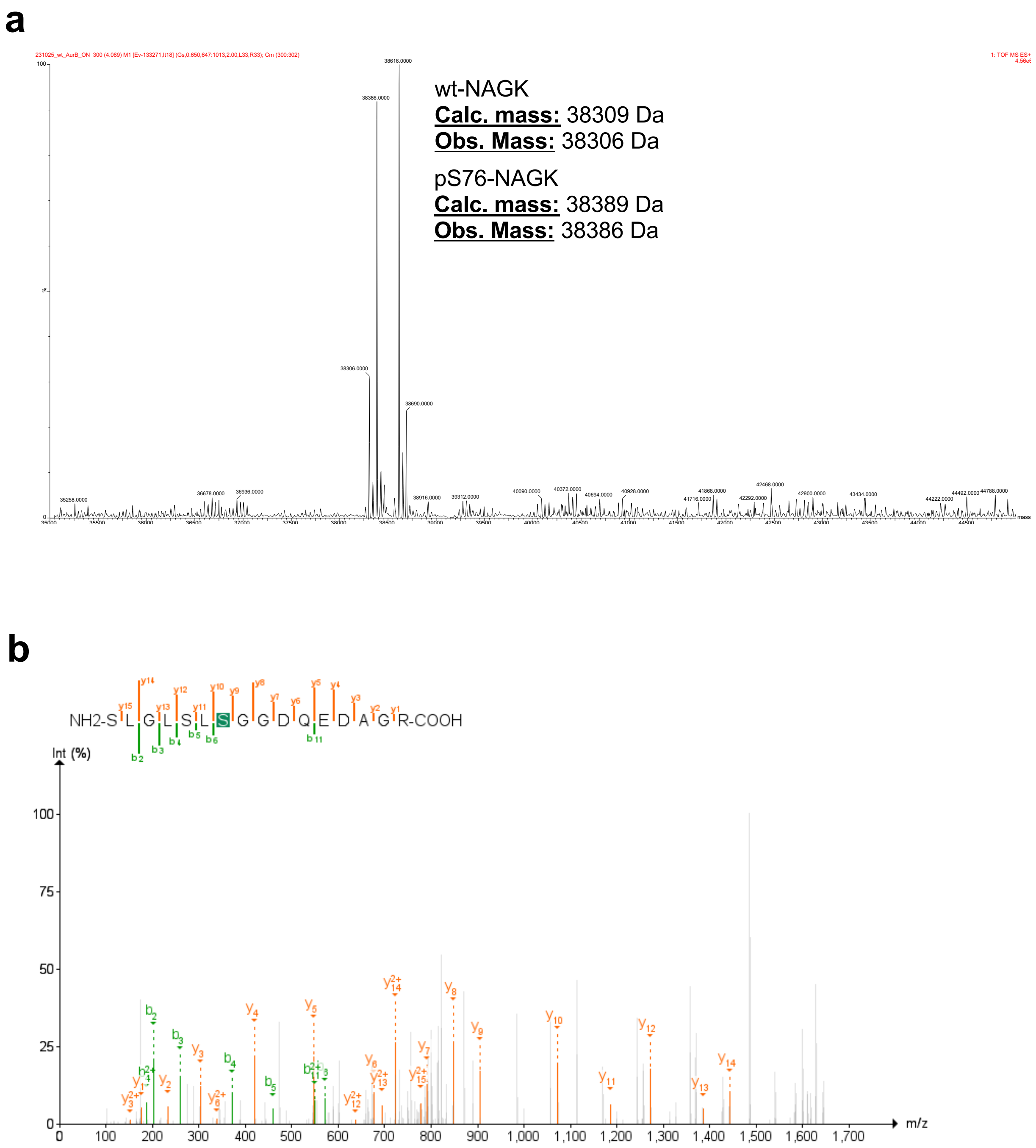

Figure S6. a) Q-TOF-MS analysis of wt-NAGK treated with AurB, indicating phosphorylation. The additional signal at 38616 Da can likely be attributed to glutathionylation of wt-NAGK. b) MS/MS analysis of wt-NAGK following treatment with AurB, localizing phosphorylation to S76.[5]

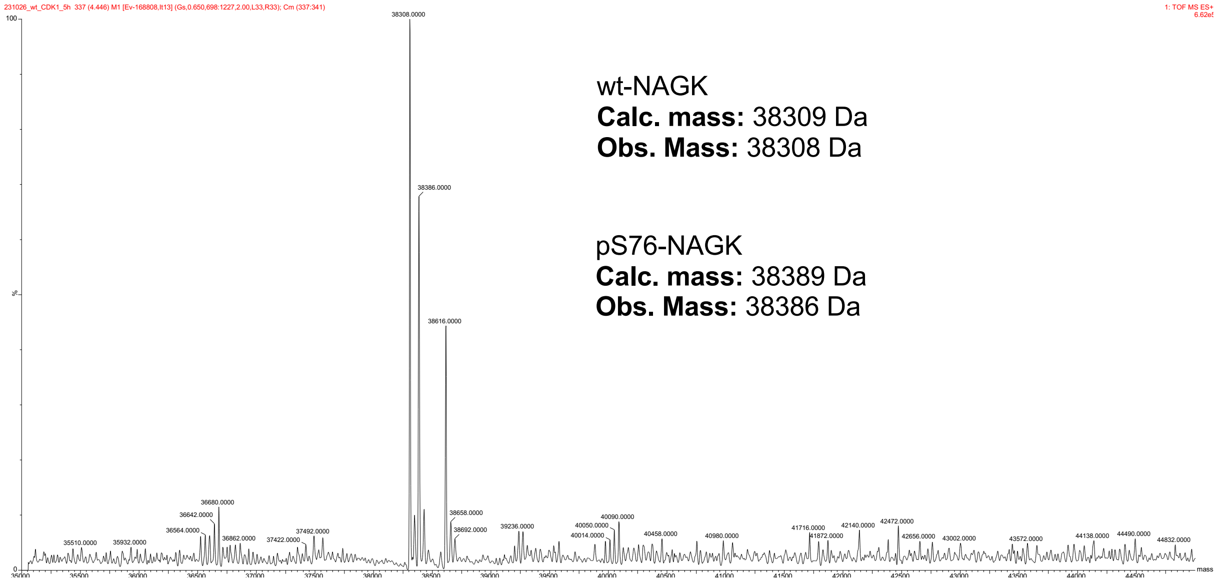

Figure S7. Q-TOF-MS analysis of wt-NAGK, treated with CDK1, indicating phosphorylation. The additional signal at 38616 Da can likely be attributed to glutathionylation of wt-NAGK.

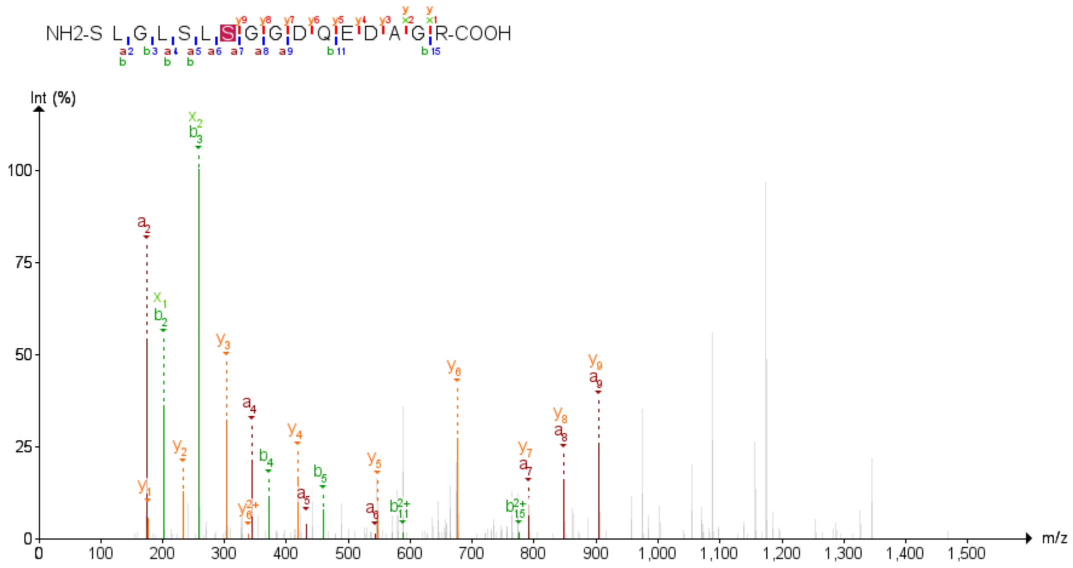

Figure S8. MS/MS analysis of ATP-treated pS76-NAGK, localizing the pyrophosphorylation site to S76.[5]

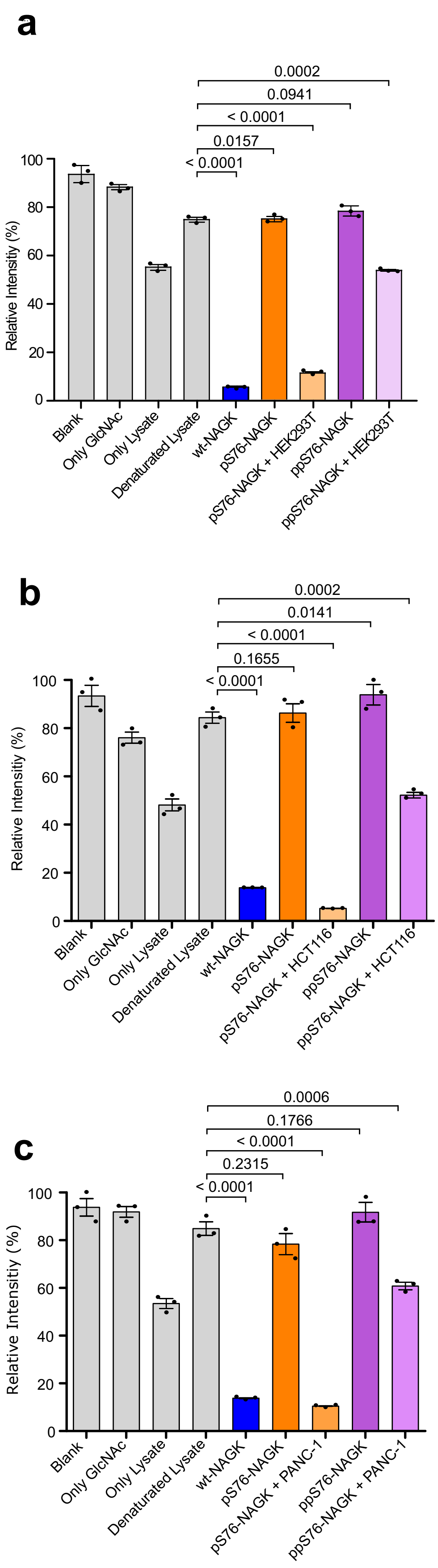

Figure S9. Biochemical validation of NAGK (wt, pS76, ppS76) after treatment with a) HEK293T lysate, b) HCT116, and c) PANC-1 lysate following by GlcNAc kinase activity assay showed regained activity of pS76-NAGK but not ppS76-NAGK indicating no pyrophosphatase activity in both cell lines. Data presented as mean ±SEM of three technical replicated (N=3). P-values were determined by unpaired t-test analysis.

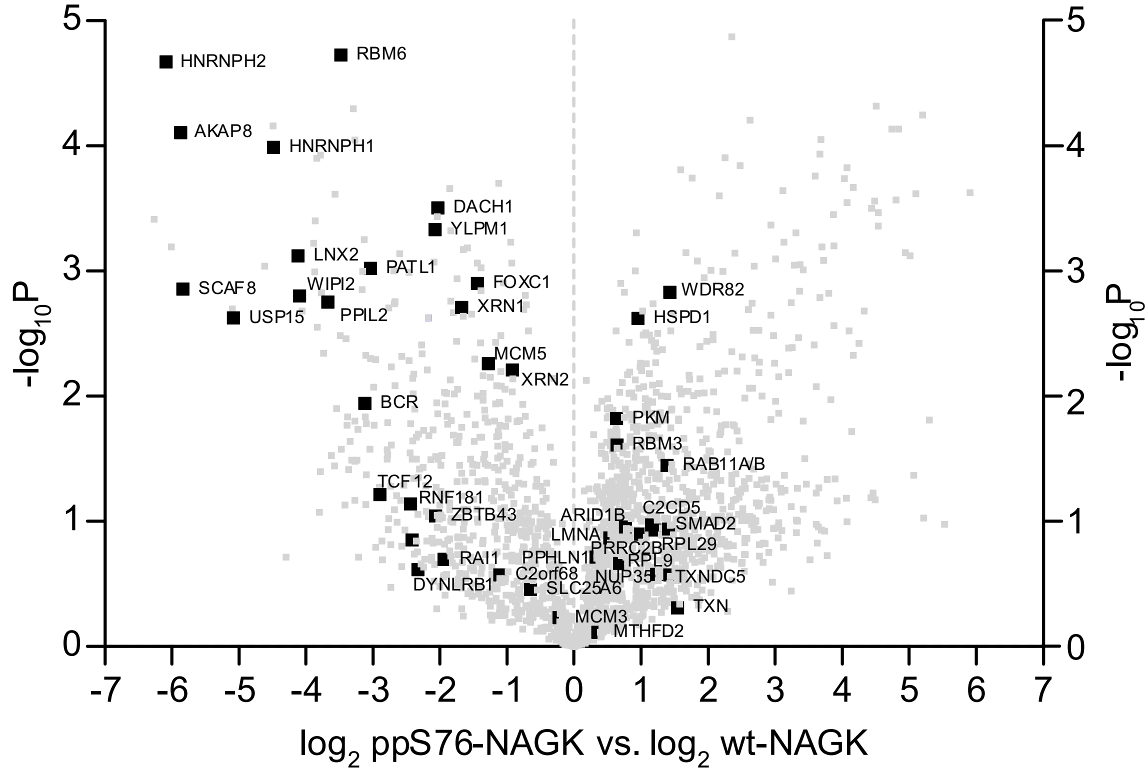

Figure S10. Volcano plot depicting LFQ values of ppS76-NAGK versus wt-NAGK after a t-test. The x-axis displays the difference of LFQ values on a $\log_2$ scale and the y-axis shows the $-\log_{10}P$ value. The left side of the plot represents a known interactor (BioGRID database) preferentially enriched with wt-NAGK. The right side of the plot represents known interactors (BioGRID database) preferentially enriched with ppS76-NAGK.[6,7,8]

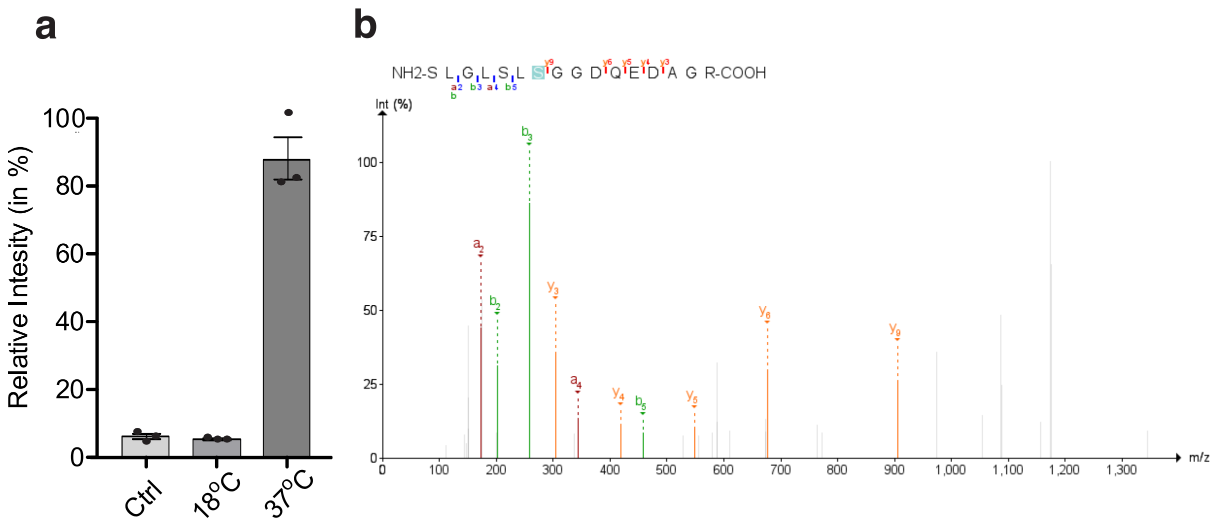

Figure S11. a) Relative quantification of in vitro pyrophosphorylation, incubating pS76-NAGK with ATP (2 mM) overnight at 18 ˚C and 37 ˚C. Ctrl sample did not contain ATP. b) MS/MS analysis revealed pyrophosphorylation occurred only at 37 ˚C incubation.

2. General Information

Cell lines

HEK293T (female human origin), HCT116 (male human origin), and PANC-1 (pancreatic carcinoma-1) cell lines were used in this study. HEK293T and HCT116 cell lines were obtained from ATCC (American Type Culture Collection). PANC-1 cell lines were kindly provided by Kathryn E. Wellen. All cells were tested for mycoplasma before use. Cells were grown in 15-cm dishes to 70-80% confluency in Dulbecco's Modified Eagle's Medium (DMEM), complemented with 10% FBS, Penicillin-Streptomycin (100 U/mL), Glutamine (2 mM) in a 5% humidified CO_2 incubator at 37 ˚C.

Chemicals and Solvents

Commercially available chemicals were purchased from Sigma-Aldrich, Alfa Aesar, Acros Organics, Strem Chemicals, TCI America, Anaspec, Carl Roth GmbH., and Iris. Dichloromethane and THF were dried by passing through an activated alumina column, and acetonitrile, DMSO, and DMF were dried by passing through a column of activated molecular sieves using a Pure Process Technology drying system.

Preparative HPLC

Preparative high-performance liquid chromatography (HPLC) was performed on a Varian system with an SD-1 prep solvent delivery system, a ProStar 325 UV-Vis detector, and a 440-LC fraction collector, using a Waters XBridge^{TM} 5 µm C18 column (19 × 150 mm).

Q-TOF-MS

High-resolution ESI-MS spectra were recorded on two different instruments: 1) Agilent 6220 TOF Accurate Mass coupled to an Agilent 1200 LC (Agilent Technologies, USA) and were measured at 35 ˚C between 100–2000 m/z. The used column was an Accucore RP-MS (30 x 2.1 mm; 2.6 µm particle size) eluted with a flow of 0.8 mL/min and the following gradient (A = H_2O + 0.1% TFA, B = MeCN + 0.1% TFA), gradient: 5% B 0–0.2 min, 5–99% B 0.2–1.1 min, 99% B 1.1–2.5 min. 2) Agilent Technologies 6230 Accurate Mass TOF LC/MS linked to Agilent Technologies HPLC 1260 Series; Column: Thermo Accucore RP-MS; Particle Size: 2.6 µM Dimension: 30 x 2.1 mm. The following gradient was used: A = H_2O + 0.1 % formic acid, B = MeCN + 0.1 % formic acid, 5% B 0.0–0.2 min, 5-99% B 0.2–1.1 min, 99% B 1.1–3.6 min, 5% B 3.6–4.9 min. Flow rate: 0.8 mL/min; UV-detection: 220 nm, 254 nm, 300 nm.

Intact protein MS

Intact proteins were analyzed using a Waters H-class instrument equipped with a quaternary solvent manager, a Waters sample manager-FTN, a Waters PDA detector, and a Waters column manager with an Acquity UPLC protein BEH C4 column (300 Å, 1.7 µm, 2.1 mm x 50 mm). Proteins were eluted at a column temperature of 80 ˚C with a flow rate of 0.3 mL/min.

The following gradient was used: A = H_2O + 0.01% formic acid, B = MeCN + 0.01% formic acid. 5–95% B 0–6 min at 40 ˚C. Mass analysis was conducted with a Waters XEVO G2-XS Q-TOF analyzer. Proteins were ionized in positive ion mode applying a cone voltage of 40 kV. Raw data was deconvoluted with MaxEnt.

2.1 Cloning, site-directed mutagenesis, expression, and purification of recombinant human NAGK

Cloning

A gene sequence encoding for human NAGK (full-length, Uniprot Q9UJ70) was purchased using Thermo Fisher's GeneArt service. The sequence was codon optimized for expression in *E. coli* and contains a NdeI (at initial ATG) and XhoI (after the stop codon) restriction site. The NAGK gene was cloned into the vector pET-21a using the NdeI and XhoI restriction sites. The resulting plasmid (pET-15b-NAGK) encodes a C-terminal His-tag and NAGK.

Site-directed mutagenesis

Site-directed mutagenesis was performed on a Bio-Rad C1000 Touch Thermal Cycler machine. Plasmid DNA harboring the corresponding NAGK ORF was extracted from an overnight culture of the pET21-NAGK vector in TB-Amp using a QIAGEN Miniprep kit. Single point mutations were installed by employing the Forward (5 – GCTGGGTGAGTAGAATCCTGCTG) and reverse (5' – ATCACGCGACCTGTCTTT) primer. 50 µl PCR reactions were performed on a Bio-Rad C1000 Touch Thermal Cycler following the NEB Phusion High-Fidelity DNA polymerase protocol using the following temperature program: 5 min 98 ˚C → 30 s 98 ˚C → 3 min 62 ˚C → Cycle to step 2 30× → 5 min 72 ˚C

Mutagenesis Primers

Forward: AGCCTGTAGGGTGGTGATCAAGAAG

Reverse: ACCACCCTACAGGCTCAGACC

Sequencing Primer

T7 Forward: 5' – TAATACGACTCACTATAG – 3'

Plasmid Transformation

Heat-shock procedure

Roughly 100 ng DNA was added to competent *E. Coli* cells and incubated on ice for 30 min. After the heat shock (40 s at 42 ˚C), the cells were kept on ice for 5 min. Subsequently, 500 µL SOC outgrowth medium was added and the cells incubated for 30 min at 37 ˚C before being plated onto LB plates supplemented with the required antibiotic, and grown overnight at 37 ˚C. Colonies were picked and inoculated for overnight cultures in LB supplemented with the required antibiotic (Ampicillin 50 µg/mL, Chloramphenicol: 25 µg/mL) out of which glycerol stocks were prepared. The obtained plasmids were extracted with QIAprep 2.0 Spin Miniprep Kit following the manufacturer's procedure and sent in for sequencing.

Electroporation procedure

Electrocompetent BL21 (DE3) ∆serB cells (25 µL) were combined with 10 ng of DNA plasmids in electroporation cuvettes. Pulsing was performed using a Gene Pulser X-cell system (BioRad) with default *E. coli* settings (Voltage: 1800 V, Capacitance: 25 µF, Resistance: 200 Ω, Gap length: 1.0 mm). Post-pulsing, pre-warmed SOC medium was added within 30 s. Electroporated cell suspensions were transferred to 5 mL tubes, incubated (37 ˚C, 200 rpm) for 1 h, and plated onto LB plates with appropriate antibiotics.

Gel Electrophoresis and Staining

Protein samples were combined with 4x Laemmli sample buffer (Bio-Rad) containing 10% β-mercaptoethanol and heated to 95 ˚C for 8 min. Following heating, the samples were cooled and consolidated by centrifugation at 5000 x g for 1 minute before loading onto Bio-Rad Mini-Protean TGX Stain-Free precast 4–20% gradient gels with 10x 30 µL wells. Gel electrophoresis was conducted using a Bio-Rad PowerPac HC 300 W power source set to 150 V for 1 h or until the loading dye migrated off the gel. Subsequently, gels underwent three 5min washes with deionized water (D.I. H_2O) and were stained for 1 h with GelCode^{TM} Blue colloidal Coomassie stain G-250 from ThermoFisher Scientific. Finally, gels were de-stained through multiple washes with D.I. water.

Protein Expression

Expression of wildtype-NAGK

E. Coli BL21 (DE3) harboring the required pET21a NAGK vector were inoculated in 10 mL LB-Amp overnight at 37 ˚C. The overnight culture was diluted to a final OD_{600} of 0.05 and grown to OD_{600} of 0.7 at 37 ˚C. The temperature was switched to 18 ˚C and expression was induced with 1 mM isopropyl β-D-1-thiogalactopyranoside (IPTG). After overnight expression at 18 ˚C the cells were harvested by centrifugation (3000 x g, 10 min, 4 ˚C) and washed with ice-cold water. The cell pellet was stored at -80 ˚C.

Expression of pS76-NAGK *via* Amber Codon Suppression

E. Coli BL21 (DE3) ΔserB harboring the required pET21a (Amp^R) NAGK and pKW1-Sep ($Camp^R$) vector were inoculated in LB-Amp-Camp overnight at 37 ˚C. The overnight culture was diluted to a final OD_{600} of 0.05 and grown to OD_{600} of 0.7 at 37 ˚C in the presence of Amp and Camp. The temperature was switched to 18 ˚C and expression was induced with 1 mM isopropyl β-D-1-thiogalactopyranoside (IPTG) and 2 mM (L)-O-phosphoserine (pH 7.0). After overnight expression at 18 ˚C the cells were harvested by centrifugation (3000 x g, 10 min, 4 ˚C) and washed with ice-cold water. The cell pellet was stored at -80 ˚C.

General protein purification procedure

The frozen cell pellet was thawed and resuspended in lysis buffer (50 mM Tris-HCl (pH 7.8), 150 mM NaCl, 50 mM Imidazole) and supplemented with lysozyme, DNase I, and 1 tablet of complete protease inhibitor (purchased from Sigma-Aldrich). After 30 min of incubation on ice, the cell extract was lysed with a microfluidizer™ LM10 at 15000 psi with five iterations. The cell debris was removed by centrifugation (30000 x g, 30 min, 4 ˚C), and the supernatant lysate was filtered (VWR vacuum filter, PES, 0.45 µm). Recombinantly expressed proteins were purified on an FPLC system (NGC Quest 10 Chromatography System, Bio-Rad). For purification, the lysate was loaded onto a Co-NTA column that was equilibrated with lysis buffer at a flow rate of 2.5 mL/min. The protein was eluted with a 0–100% gradient of elution buffer (50 mM Tris-HCl (pH 7.8), 150 mM NaCl, 500 mM Imidazole). The volume of the fractions that contained the desired protein was reduced by spin filtration through a 10 kDa cut-off filter and dialyzed overnight against dialysis buffer (50 mM Tris-HCl (pH 7.8), 150 mM NaCl, 1 mM DTT, and 10% Glycerol). Protein concentrations were determined using a PierceTM BCA Protein Assay Kit. Recombinantly expressed proteins were purified on an FPLC system (NGC Quest 10 Chromatography System, Bio-Rad).

wt-NAGK

Yield: 33 mg/L.

Sequence:

AAIYGGVEGGGTRSEVLLVSEDGKILAEADGLSTNHWLIGTDKCVERINEMVNRAKRKAGVD
PLVPLRSLGLSL**S**GGDQEDAGRILIEELRDRFPYLSESYLITTDAAGSIATATPDGGVVLISGT
GSNCRLINPDGSESGCGGWGHMMGDEGSAYWIAHQAVKIVFDSIDNLEAAPHDIGYVKQA
MFHYFQVPDRLGILTHLYRDFDKCRFAGFCRKIAEGAQQGDPLSRYIFRKAGEMLGRHIVAV
LPEIDPVLFQGKIGLPILCVGSVWKSWELLKEGFLLALTQGREIQAQNFFSSFTLMKLRHSSA
LGGASLGARHIGHLLPMDYSANAIAFYSYTFSLEHHHHHH

pS76-NAGK

Yield: 7.5 mg/L.

Sequence:

AAIYGGVEGGGTRSEVLLVSEDGKILAEADGLSTNHWLIGTDKCVERINEMVNRAKRKAGVD
PLVPLRSLGLSL**pS**GGDQEDAGRILIEELRDRFPYLSESYLITTDAAGSIATATPDGGVVLISG
TGSNCRLINPDGSESGCGGWGHMMGDEGSAYWIAHQAVKIVFDSIDNLEAAPHDIGYVKQ
AMFHYFQVPDRLGILTHLYRDFDKCRFAGFCRKIAEGAQQGDPLSRYIFRKAGEMLGRHIVA
VLPEIDPVLFQGKIGLPILCVGSVWKSWELLKEGFLLALTQGREIQAQNFFSSFTLMKLRHSS
ALGGASLGARHIGHLLPMDYSANAIAFYSYTFSLEHHHHHH

2.2 Protocol for protein pyrophosphorylation[9]

The storage buffer for all protein aliquots used in pyrophosphorylation reactions was removed and exchanged for Milli-Q H_2O by at least 5 cycles of spin desalting. Amicon® Ultra Centrifugal Filters from Millipore Sigma with capacities of 15 mL or 500 µL were used for the concentration and desalting of protein solutions. Steps that call for heating and agitation of protein samples were performed in an Eppendorf™ ThermoMixer®, equipped with a heated lid. Solutions made with DMA were passed through a 0.45 µm PTFE syringe filter before use. Photochemical reactions were conducted using an Atlas Photonics Lumos 43 light source with an optical output of 200 mW/cm^2.

Protocol for protein pyrophosphorylation and refolding

A solid sample of phosphorimidazolide (P-imidazolide) reagent was supplemented with DMA containing 340 mM $ZnCl_2$ to yield a 68 mM P-imidazolide solution. Subsequently, a Protein low binding 0.5 mL tube containing a 5.0 µL aliquot of 10.0 µg/µL NAGK in Milli-Q H_2O received 45.0 µL of the freshly prepared P-Imidazolide solution. The resultant clear yellow solution (comprising 61.2 mM P-imidazolide, 26 µM protein, and 306 mM $ZnCl_2$, in a 1:9 ratio of H_2O to DMA) underwent incubation at 45 ˚C with 1000 rpm shaking for 21 h. A 50 µL aliquot was quenched by dilution into 450 µL of solubilization buffer (50 mM Tris-HCl (pH 8.0), 6.0 M Guanidine chloride. 0.2% CHAPS, 10 mM DTT) and further incubated for 2 h at 37 °C. The solution was treated with refolding buffer (composed of 50 mM Tris-HCl pH= 8.0, 250 mM NaCl, 0.3 mM GSSG, 3 mM GSH, 10 mM EDTA, 0.2% CHAPS, 0.1 M Arginine*HCl, 1 mM GlcNAc) and dialyzed overnight at 4 ˚C. Subsequently, the samples underwent concentration via multiple rounds of centrifugation in a 30 kDa MWCO centrifuge filter unit with a 15 mL capacity (20 min, 3,214 x *g*, 17 ˚C) to obtain the concentrated protein with a yield of 10%. Finally, NAGK was subjected to 360 nm light irradiation for 1 h and subsequently analyzed by Q-TOF-MS.

Circular Dichroism (CD) Spectroscopy:

CD spectra were obtained on a Jasco J-720 spectropolarimeter using Jasco J-700 series control driver software, version 1.08.00 [Build 3]. Data was analyzed with Jasco Spectra Analysis software, version 1.53.04 [Build 1]. Spectra were taken in a Hellma 100-QS Quartz SUPRASIL® Cuvette with a 1.0 mm path length. Samples were prepared by exchanging into Phosphate buffered Fluoride (PBF) buffer (consisting of 154 mM NaF, and 10 mM Na_2HPO_4 in Milli-Q H_2O adjusted to pH 7.4, made with 99.99% NaF (trace metals basis, from Sigma-Aldrich)) by at least five cycles of spin desalting (18,000 x *g,* 20 min, rt) in 500 µL 10 kDa MWCO centrifugal filters, by diluting the sample to 500 µL with PBF between spins. Sample protein concentrations were adjusted to 10 µM as determined by UV absorbance at 280 nm.

2.3 General Protocol for NAGK biochemical assays

NAGK kinase assay

GlcNac kinase activity analyzed by NADH consumption assay:

Purified NAGK (10 nM) was added to a reaction mixture containing 50 mM HEPES, 2 mM ATP, 2.2 mM ATP, 0.2 mM β-NADH, 1.1 mM PEP, 10 mM $MgCl_2$ 10 units lactic dehydrogenase and 7 units pyruvate kinase to a final volume of 100 µL. The reaction was vortexed immediately and a decrease in absorbance of NADH at 340 nm was then recorded for 5 min. UV signals were read out with a TECAN Infinite M Plex plate reader.

GlcNac kinase activity analyzed by Kinase-Glo® assay:

Purified NAGK (1 nM–1000 nM) was added to a reaction mixture containing 100 µM ATP, 50 mM HEPES, 100 mM NaCl, 10 mM $MgCl_2$, 1 mM DTT, and 70 µM GlcNAc. After 1 h at 37 ˚C, *Promega* Kinase-Glo Plus® reagent was added and the luminescence read out with a *Tecan* Infinite M Plex reader using 100 ms exposure time after 10 min of equilibration. Luminescence signals were read out with a TECAN Infinite M Plex plate reader.

Autopyrophosphorylation assay

pS76-NAGK (10 µM) was incubated in kinase buffer (50 mM HEPES pH= 7.4, 100 mM NaCl, 10 mM $MgCl_2$, 200 µM—2 mM ATP, 1 mM DTT) at 37 ˚C overnight. Subsequently, the sample was digested by trypsin in solution and analyzed by MS/MS. Relative quantification was determined by the Software FreeStyle™ 1.7 integrating the MS1 intensities relative to the background.

Mammalian cell lysate-based stability assay

pS76-NAGK and ppS76-NAGK (10 µM) were incubated in 50 mM HEPES (pH 7.5), 100 mM NaCl, 10 mM $MgCl_2$, 1 mM $MnCl_2$, 1 mM DTT and 100 µg cell lysate overnight at 37 ˚C. Subsequently, the reaction was applied to the NAGK activity assay using Kinase-Glo® assay and analyzed with a TECAN Infinite M Plex plate reader.

2.4 General information for interactome analysis

HEK293T cell culture and lysate processing

HEK293T cells were grown in 15-cm dishes to 70–80% confluency in Dulbecco's Modified Eagle's Medium (DMEM), complemented with 10% FBS, Penicillin-Streptomycin (100 U/mL) and Glutamine (2 mM). Cells were washed twice with ice-cold DPBS (10 mL) and lysed by sonication (IKA Labortechnik, U200S control, 0.5 cycles, 50% intensity, 5 rounds) in 50 mM TBS buffer. supplemented with phosphatase and protease inhibitors (Roche PhosStopTM and cOmpleteTM EDTA-free protease inhibitor cocktail). The cells were scraped off, transferred to protein low-binding microcentrifuge tubes, and incubated on ice for 10 min. The lysate was

then centrifuged at 4 ˚C for 10 min at 17,900 x g. The supernatants were combined and lysate protein concentration was determined using Pierce™ Coomassie (Bradford) protein-assay-kit.

Affinity Capture Experiments for Proteomic Analysis

All steps were conducted at 4 ˚C. A suspension of Nickel-beads (50 µL) was washed three times with 1 mL Milli-Q H_2O and three times with 1 mL TBS Buffer (50 mM Tris-HCl pH= 7.5, 150 mM NaCl.) supplemented with 2 mM $MgCl_2$ and 2 mM $MnCl_2$. Subsequently, 25 µg of recombinant NAGK (wildtype, S76pS, S76ppS) in 100 µL TBS Buffer was added to the beads and incubated with constant rotation for 1 h at 4 ˚C. Beads were centrifuged at 2000 x *g* and the supernatant was discarded. The beads were washed three times with 1 mL TBS buffer and 1 mg of HEK293T cell lysate in TBS buffer was added to the beads and incubated for 3 h at 4 ˚C under rotation. Upon this time, the beads were centrifuged at 2000 x *g*, the supernatant was discarded and then they were washed three times with 1 mL TBS buffer. Lastly, proteins were incubated with elution buffer (50 mM Tris-HCl (pH 7.5), 150 mM NaCl, 500 mM Imidazole) for 1 h at 4 ˚C under constant rotation. After centrifugation at 2000 x *g*, the supernatant was collected and lyophilized.

In solution tryptic digestion

Lyophilized samples were resolubilized in 100 µl Buffer (50 mM TEAB, 6 M Urea) and diluted to 2 M Urea. Samples were subsequently reduced and alkylated with 5 mM TCEP and 20 mM Iodoacetamide (IAA) at 37 ˚C for 1 h. Trypsin was added at an enzyme-to-protein ration of 1:100 (w/w) to digest overnight at 37 ˚C. Trypsin was then quenched with 1% FA (endconcentration) and centrifuged for 10 min at 20000 x g. The supernatant was collected and desalted using Sep-PAK C18 cartridges and lyophilized. Peptides were quantified by using BCA quantification.

Liquid Chromatography and mass spectrometry

LC-MS/MS analysis was performed using an UltiMate 3000 RSLC nano-LC system coupled on-line to an Orbitrap Fusion or Lumos mass spectrometer (Thermo Fisher Scientific). For sample loading a PepMap C-18 trap-column (Thermo Fischer Scientific) of 0.075 mm ID x 50 mm length, 3 µm particle size, and 100 Å pore size was used. The loading mobile phase A contained 1% acetonitrile and 0.05% TFA acid in water, and mobile phase B 0.05% TFA acid in acetonitrile. Reversed-phase separation was performed using a 50 cm analytical column (in-house packed with Poroshell 120 EC-C18, 2.7 µm, Agilent Technologies) with mobile phase A containing 0.1% formic acid in water, and mobile phase B 0.1% formic acid in acetonitrile. The gradient started with 4% buffer B reaching 40% buffer B in 95 min, with a total run time of 120 min including column wash and equilibration. MS1 scans were performed in the orbitrap using

120000 resolution; MS2 scans were acquired in the ion trap with an AGC target of 10000 and maximum injection time of 35 ms, charge state 2-4 enable for MS2.

Identification, quantification, and statistics of proteomics data

Raw data were analyzed and processed using MaxQuant software version 1.6.2.6a. Analysis was done with standard settings. Search parameters included two missed cleavage sites, fixed cysteine carbamidomethyl modification, and variable modifications including methionine oxidation and N-terminal protein acetylation. The peptide mass tolerance was 4.5 ppm for MS scans and 20 ppm for MS/MS scans. The match between-runs option was enabled. Database search was performed using Andromeda against the Human UniProt/Swiss-Prot database (October 2016) with common contaminants. The false discovery rate (FDR) was set to 1% at both the peptide and protein levels. Protein quantification was done based on razor and unique peptides. Label-free quantification was enabled. Bioinformatic analysis was carried out using Perseus software version 1.6.7.0. Proteins were filtered to exclude reverse database hits, potential contaminants, and proteins only identified by site. Proteins were further filtered by rows, requiring a valid value for at least two proteins out of four technical replicates. Data was imputed using Perseus default parameters, 0.3 widths, and 1.8 downshift. Volcano plots were generated using a t-test (number of randomizations: 250).

2.5 Chemical Synthesis and Characterization

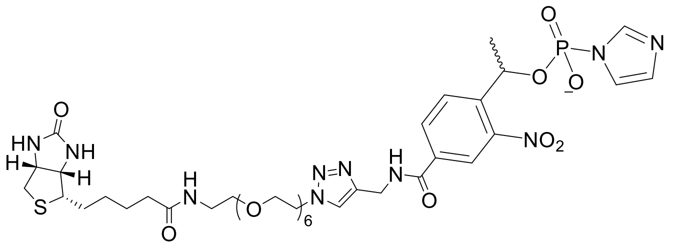

Biotin-PEG$_6$-trayole-NPE-(1H-imidazol-1-yl)phosphonate was synthesized as previously described.[9] Spectral data matches previously reported values.

3. Q-TOF-MS spectra

wt-NAGK

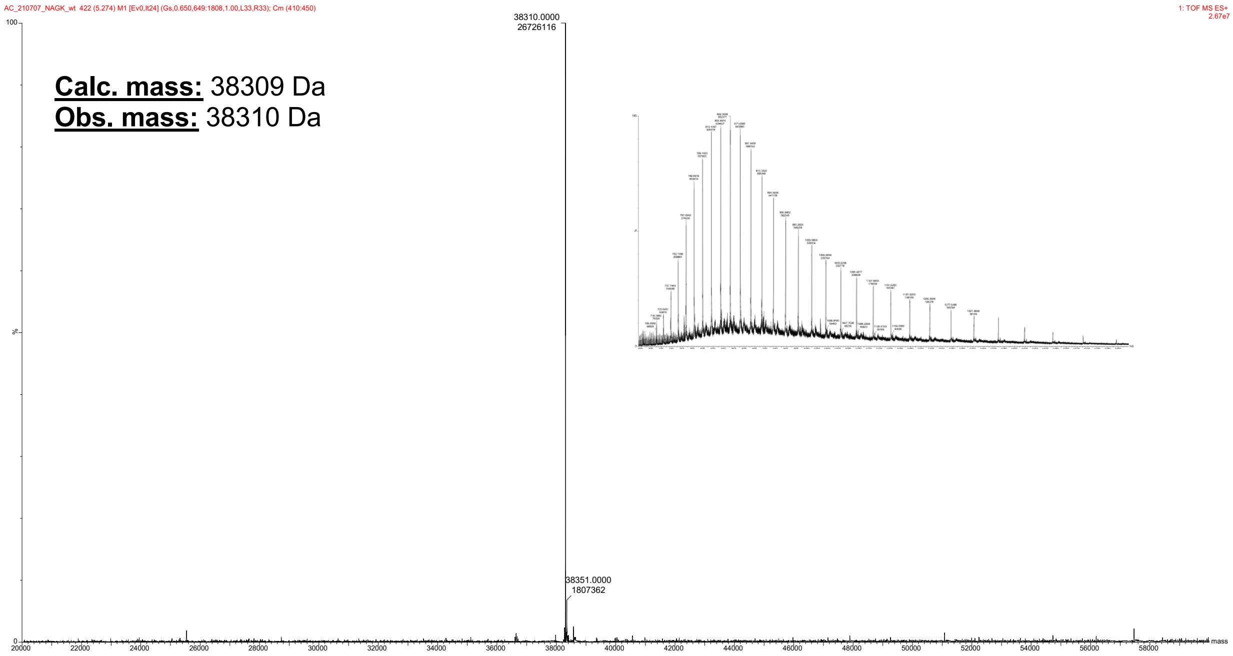

pS76-NAGK

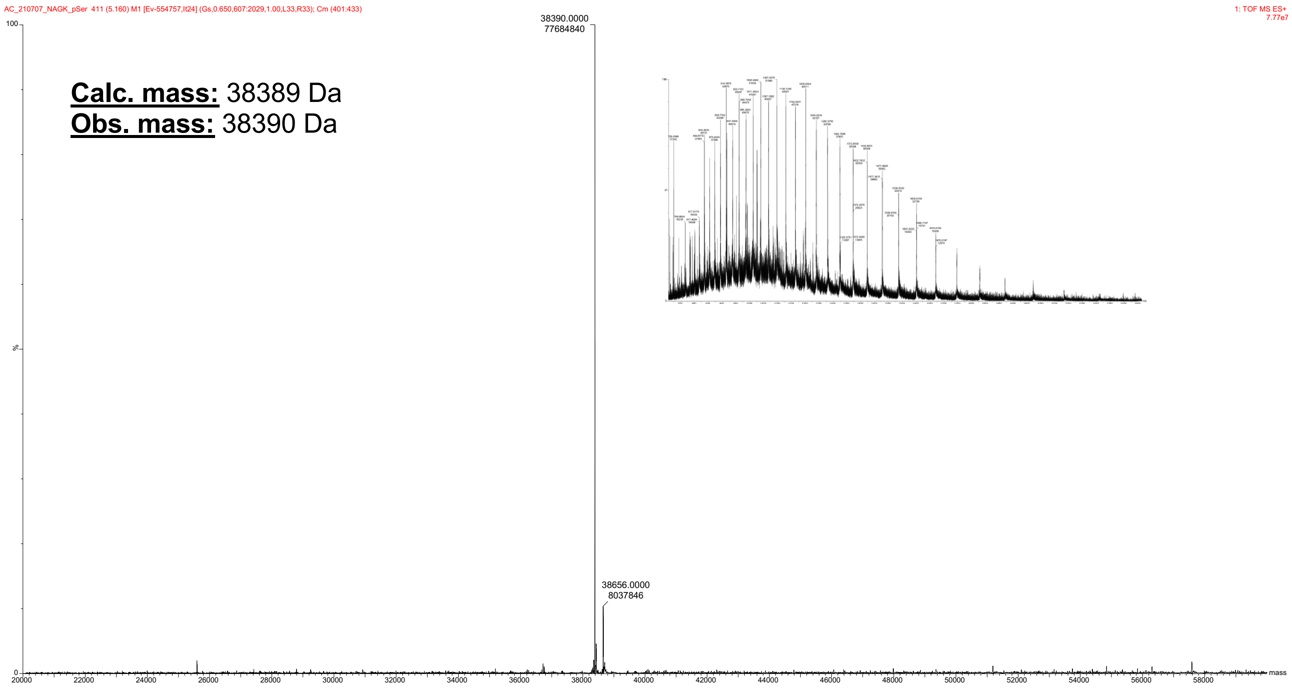

R-ppS76-NAGK

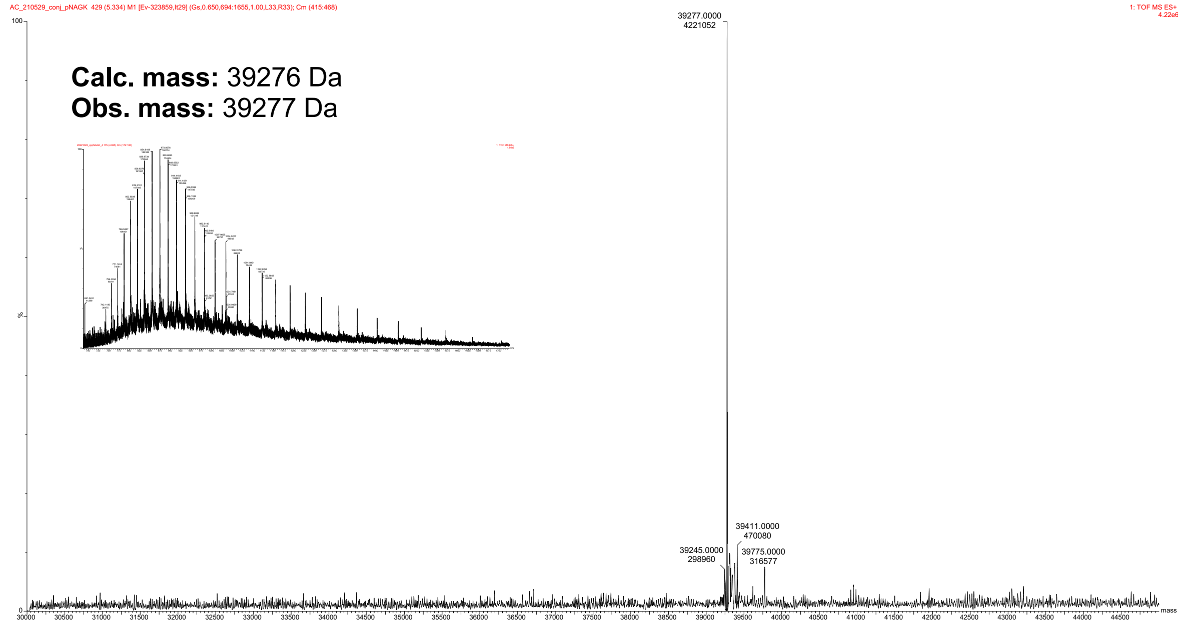

ppS76-NAGK

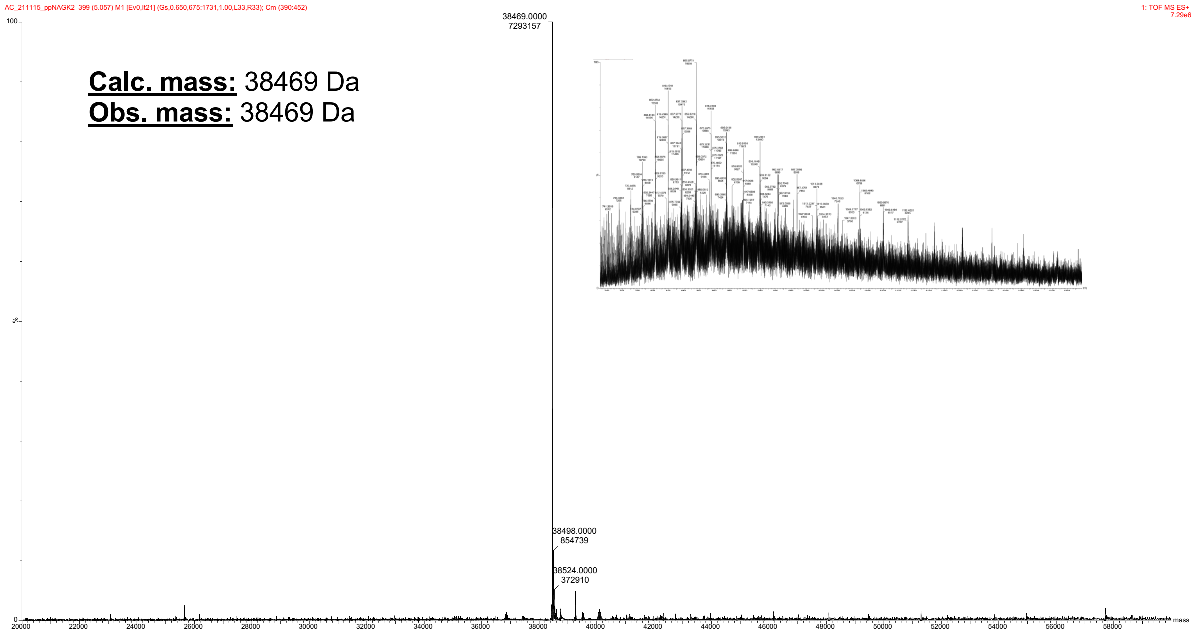

ppS76-NAGK treated with HEK293T lysate

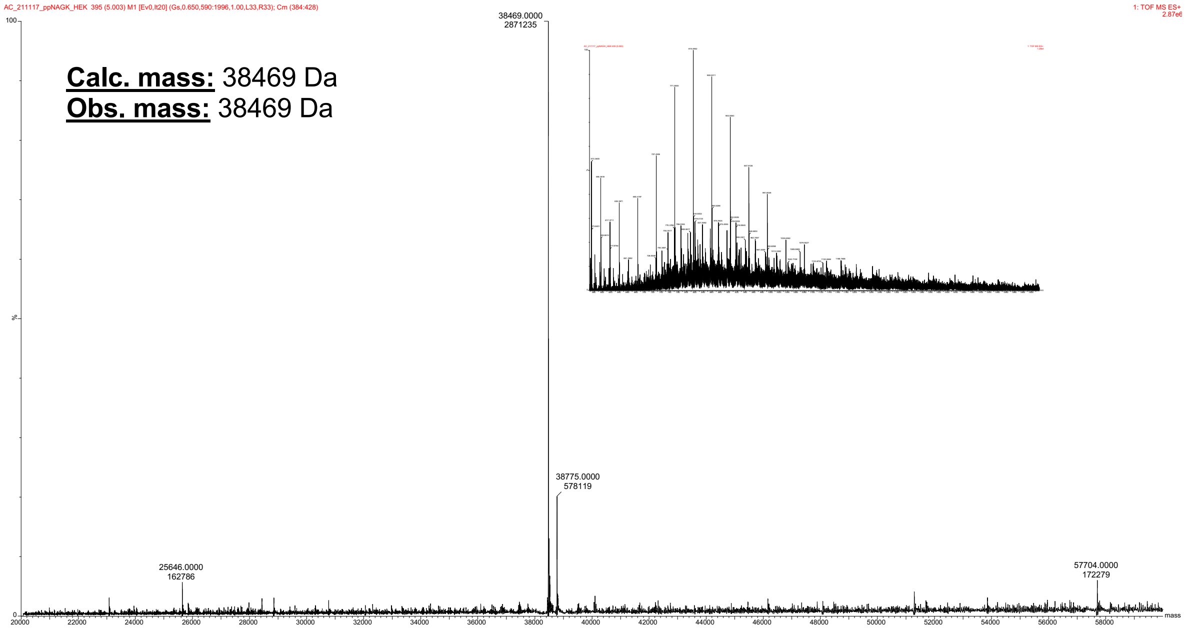

The additional signal at 38775 Da can likely be attributed to glutathionylation of ppS76-NAGK.

pS76-NAGK treated with HEK293T lysate

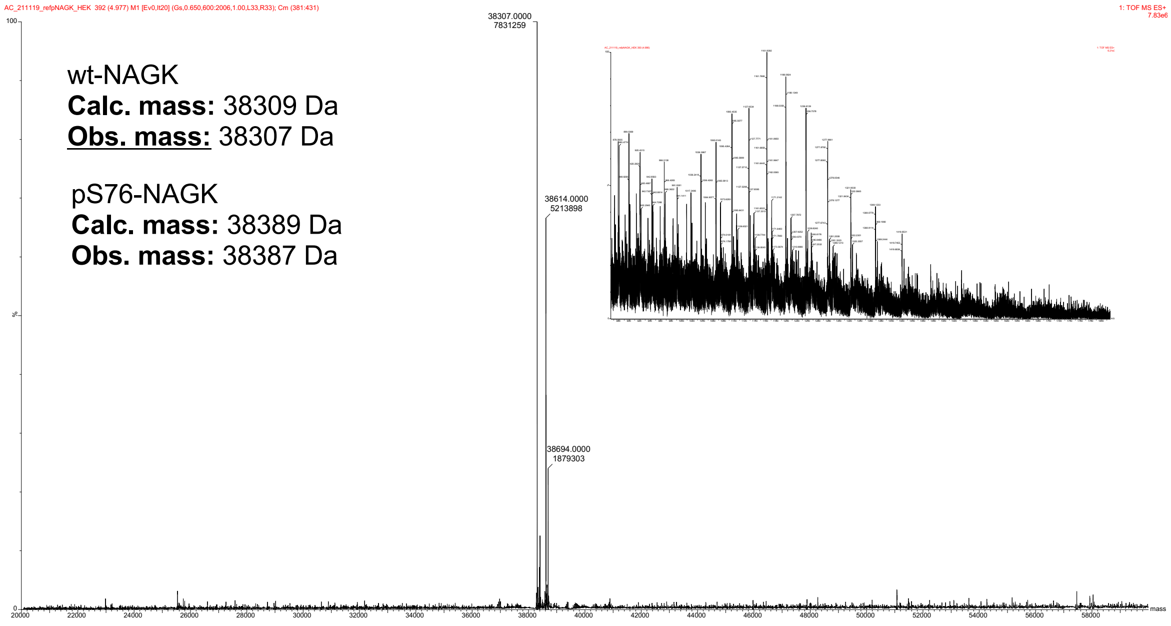

The additional signal at 38616 Da can likely be attributed to glutathionylation of pS76-NAGK.

wt-NAGK treated with AurB

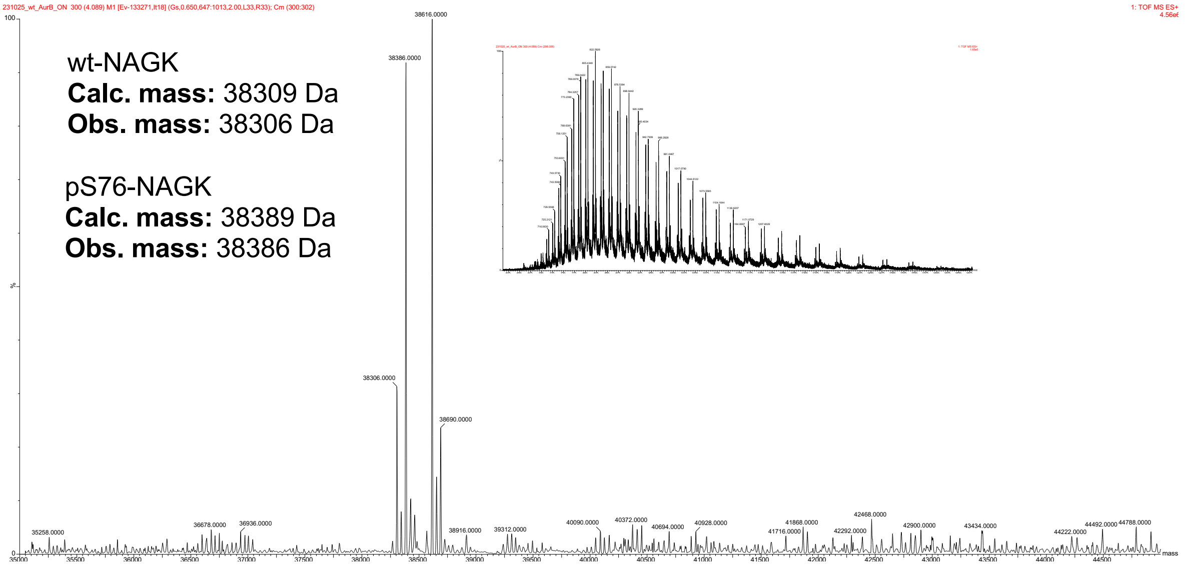

The additional signal at 38616 Da can likely be attributed to glutathionylation of wt-NAGK.

wt-NAGK treated with CDK1

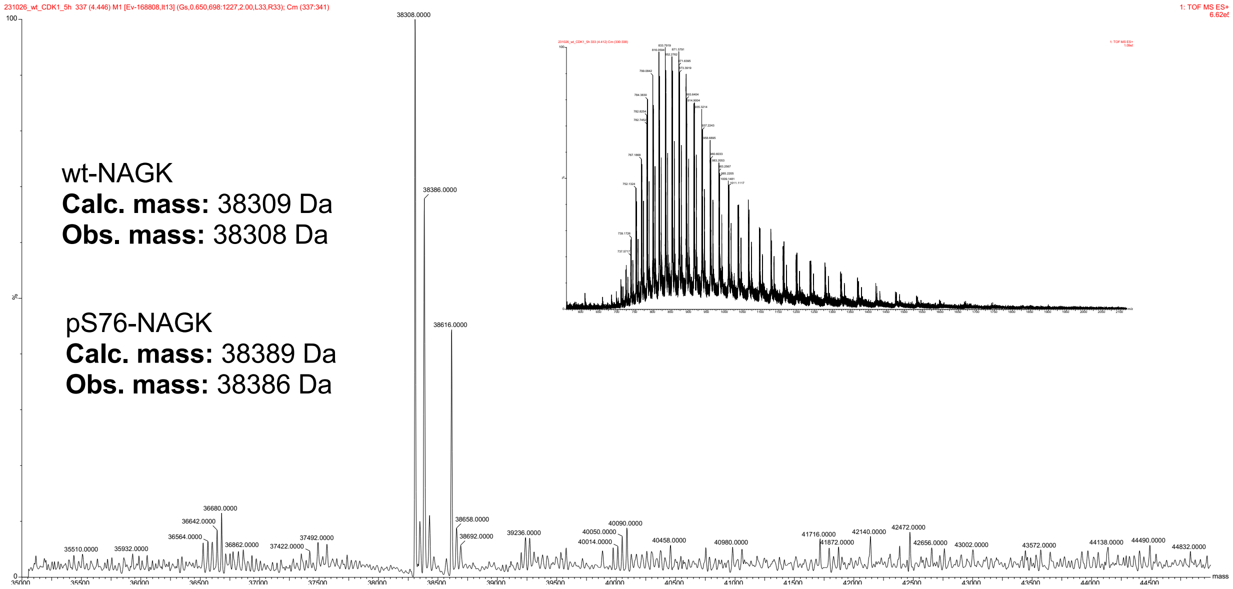

The additional signal at 38616 Da can likely be attributed to glutathionylation of wt-NAGK.

4. Reference

[1] Weihofen, W.A., Berger, M., Chen, H., Saenger, W., Hinderlich, S. Structures of Human N-Acetylglucosamine Kinase in Two Complexes with N-Acetylglucosamine and with ADP/Glucose: Insights into Substrate Specificity and Regulation. J Mol Biol, **2006**, 364 (3) 388-399.

[2] Rogerson, D. T.; Sachdeva, A.; Wang, K.; Haq, T.; Kazlauskaite, A.; Hancock, S. M.; Huguenin-Dezot, N.; Muqit, M. M. K.; Fry, A. M.; Bayliss, R.; and Chin, J. W. Efficient genetic encoding of phosphoserine and its nonhydrolyzable analog. Nature Chemical Biology, **2015**, 11 (7), 496—503.

[3] Yamaguchi, H.; Miyazaki, M. Refolding techniques for recovering biologically active recombinant proteins from inclusion bodies. Biomolecules, **2014**, 4 (1), 235—251.

[4] Nabiel, A.; Yosua, Y; Sriwidodo, S.; Maksum, I. P. Overview of refolding methods on misfolded recombinant proteins from Escherichia coli inclusion bodies. Journal of Applied Biology & Biotechnology, **2023**, 11 (3), 47—52.

[5] Kong, A. T., Leprevost, F. V., Avtonomov, D. M., Mellacheruvu, D., and Nesvizhskii, A. I. MSFragger: ultrafast and comprehensive peptide identification in mass spectrometry-based proteomics. Nature Methods, **2017**, 14 (5), 513—520.

[6] Oughtred, R.; Rust, J.; Chang, C.; Breitkreutz, B.; Stark, C.; Willems, A.; Boucher, L.; Leung, G.; Kolas, N.; Zhang, F.; Dolma, S.; Coulombe-Huntington, J.; Chatr-aryamontri, A.; Dolinski, K.; and Tyers, M. The BioGRID database: A comprehensive biomedical resource of curated protein, genetic, and chemical interactions. Protein Science, **2021**, 30 (1), 187—200.

[7] Huttlin, E. L.; Ting L.; Bruckner R. J.; Gebreab, F.; Gygi, M. P.; Szpyt, J.; Tam, S.; Zarraga, G.; Colby, G.; Baltier, K.; Dong, R.; Guarani, V.; Vaites, L. P.; Ordureau, A.; Rad, R.; Erickson, B. K.; Wuehr, M.; Chick, J.; Zhai, B.; Kolippakkam, D.; Mintseris, J.; Obar, R. A.; Harris, T.; Artavanis-Tsakonas, S.; Sowa, M. E.; Camilli, P.; Paulo, J. A.; Harper, J. W.; Gygi, S. P. The BioPlex Network: A Systematic Exploration of the Human Interactome. Cell, **2015**, 162 (2), 425—440.

[8] Huttlin, E. L.; Bruckner, R. J.; Paulo, J. A.; Cannon, J. R.; Ting, L.; Baltier, K.; Colby, G.; Gebreab, F.; Gygi, M. P.; Parzen, H.; Szpyt, J.; Tam, S.; Zarraga, G.; Pontano-Vaites, L.; Swarup, S.; White, A. E.; Schweppe, D. K.; Rad, R.; Erickson, B. K.; Obar, R. A.; Guruharsha, K. G.; Li, K.; Artavanis-Tsakonas, S.; Gygi, S. P.; Harper, J. W. Architecture of the human

interactome defines protein communities and disease networks. Nature, **2017**, 545 (765), 505—509.

[9] Marmelstein, A. M.; Morgan, J. A. M.; Penkert, M.; Rogerson, D. T.; Chin, J. W.; Krause, E.; and Fiedler, D. Pyrophosphorylation via selective phosphoprotein derivatization. Chemical Science, **2018**, 9 (27), 5929—5936.
